# Evolutionary engineering of *Geobacillus thermoleovorans* for growth on adipic acid and 1,4-butanediol

**DOI:** 10.1007/s00253-026-13836-8

**Published:** 2026-05-02

**Authors:** Leonie Op de Hipt, Amelie Jäger, Tom Luthe, Volkan Julio Cevik, Angela Kranz, Benedikt Wynands, Julia Frunzke, Björn Usadel, Nick Wierckx

**Affiliations:** 1 Institute of Bio- and Geosciences IBG-1: Biotechnology and Bioeconomy Science Center (BioSC), Forschungszentrum Jülich, Jülich, Germany; 2 Institute of Bio- and Geosciences IBG-4: Bioinformatics, CEPLAS, and Bioeconomy Science Center (BioSC), Forschungszentrum Jülich, Jülich, Germany; 3 Institute of Microbial Interactions, Faculty of Mathematics and Natural Sciences, Heinrich Heine University Düsseldorf, Düsseldorf, Germany; 4 Institute for Biological Data Science, Faculty of Mathematics and Natural Sciences, CEPLAS, Heinrich Heine University Düsseldorf, Düsseldorf, Germany; 5 Department of Translational and Computational Infection Research (TRACiR), Medical Faculty, Ruhr University Bochum, Bochum, Germany

**Keywords:** Metabolic engineering, Adaptive laboratory evolution, Transcriptomics, Plastic upcycling, PBAT, Biodegradable materials, Thermophilic bacteria, Circular economy, Consolidated bioprocessing

## Abstract

**Abstract:**

The plastic pollution crisis urges innovative recycling solutions. Promising approaches especially for polyester-containing wastes include enzymatic hydrolysis and microbial upcycling. For efficient enzymatic hydrolysis of polyesters, elevated temperatures (70–80 °C) are required, necessitating thermophilic microbial chassis for consolidated bioprocessing (CBP). In this study, we engineered *Geobacillus thermoleovorans* through adaptive laboratory evolution (ALE) for robust growth on adipic acid (AA) and 1,4-butanediol (BDO), two relevant monomers for example derived from poly(butylene adipate-*co*-terephthalate) (PBAT), enabling growth rates of up to 0.10 h^−1^ on AA and 0.13 h^−1^ on BDO. Based on a high-quality annotated genome sequence of the wild type, genomic mutations and gene expression levels were characterized in mutants grown on the respective substrates compared to glucose. For BDO, an alcohol dehydrogenase (Gth_001044) and an aldehyde dehydrogenase (Gth_001082) were identified to be likely responsible for its oxidative degradation. AA uptake appears to be mediated by a dicarboxylate transporter (Gth_003270), followed by CoA activation and β-oxidation involving a CoA transferase (Gth_003192) and several upregulated CoA-family dehydrogenases. To demonstrate applicability of these strains in plastic upcycling, they were co-cultivated with PBAT as the sole carbon source in combination with the cutinase HiC for PBAT hydrolysis. This resulted in growth on the released AA and BDO. Given the potential to purify the remaining terephthalate (TA), this approach highlights the feasibility of selective monomer valorization in bioprocesses. Additional ALE enabled co-utilization of AA and BDO by a single strain and improved AA consumption at lower concentrations, underscoring the strains’ adaptability and high potential for plastic upcycling applications.

**Key points:**

• *G. thermoleovorans evolved for robust growth on adipate and 1,4-butanediol at 60 °C.*

• *Genome and transcriptome analyses revealed underlying pathways and enzymes involved.*

• *Co-cultivation of the evolved strains on PBAT with HiC as the sole carbon source.*

**Supplementary Information:**

The online version contains supplementary material available at 10.1007/s00253-026-13836-8.

## Introduction

The global plastic crisis has emerged as one of the pressing environmental challenges of our time. Since the mid-twentieth century, plastics have become integral to modern society due to their versatility, durability, and cost-effectiveness (Lomwongsopon and Varrone 2022). This led to an enormous increase in production with 431 million tons in 2019 alone (OECD 2022). Given the linear nature of the plastic economy and insufficient waste collection, an estimated 22 million tonnes of plastic waste entered the environment in that same year (OECD 2022). Therefore, the development of new recycling strategies is essential to reduce pollution and enhance waste management.

In this context, great efforts have been made to develop cheap and robust catalysts allowing plastic depolymerization with subsequent monomer recovery for closed-loop recycling (Ellis et al. 2021; Ismail et al. 2024; Tournier et al. 2020). In particular, enzymes have high potential for hydrolysis of amorphous or semi-crystalline polyesters at temperatures in the range of or above their glass transition temperature (*T*_g_) (Tournier et al. 2023). However, even for biodegradable polymers such as poly(butylene adipate-*co*-terephthalate) (PBAT, *T*_g_ = − 30 °C), enzymatic hydrolysis is considerably more efficient at 70 °C compared to lower temperatures in the mesophilic range (30 °C) (Op de Hipt et al. 2026). Extensive research is conducted focusing on the optimization of known enzymes through protein engineering or the identification of new candidate enzymes (Austin et al. 2018; Avilan et al. 2023; Branson et al. 2024; Herbert et al. 2022; Knott et al. 2020; Sonnendecker et al. 2022; Tournier et al. 2023; Wei et al. 2022). Promising examples for the depolymerization of polyesters are cutinases such as the leaf compost cutinase (LCC) or the commercially available cutinase from *Humicola insolens* (HiC) that is applied in this study (Castro et al. 2019; Tournier et al. 2020, 2023). However, their application on complex polymers and/or mixed plastic wastes results in hydrolysates with a variety of monomers and oligomers. This heterogeneity is a challenge in downstream purification where it can impede specific and efficient monomer recovery.

The use of hydrolysates as feedstock for microorganisms that can metabolize either all or specifically difficult-to-isolate monomers is a promising alternative for upcycling approaches or to facilitate downstream processing of the remaining monomer(s) (Tiso et al. 2022; Wierckx et al. 2015). Ideally, different monomers are funneled into the microbe’s central carbon metabolism, allowing their utilization as carbon and energy sources for the biosynthesis of one upcycled product (Borchert et al. 2022; Ellis et al. 2021; Linger et al. 2014; Sullivan et al. 2022; Tiso et al. 2022).

For this purpose, the well-studied bacterium *Pseudomonas putida* KT2440 has been metabolically engineered to enable bio-upcycling of plastic monomers (Ackermann et al. 2021, 2024; de Witt et al. 2025; Li et al. 2019, 2020; Werner et al. 2025). However, due to the discrepancy between the temperature optimum of the enzymatic polyester hydrolysis (70–80 °C) and that of the mesophilic microbes (4–42 °C) (Moore 2005), the bioprocess design thus far has mostly been limited to three separate process steps: (1) enzyme production, (2) enzymatic plastic hydrolysis and (3) microbial conversion of the resulting monomers (Tiso et al. 2021; Welsing et al. 2025). These separate depolymerization and conversion steps have higher spatial and energy requirements, consume more acids and bases, and produce more salt waste, all of which add considerable processing costs (Ernst et al. 2024; Singhania et al. 2022). Also, high monomer concentrations in the hydrolysate can lead to product inhibition of the enzymes on the one hand and substrate toxicity for the strains on the other, which would further reduce process efficiency. These limitations can potentially be alleviated by combining all steps into a single consolidated bioprocess (Ellis et al. 2021). However, for successful consolidated bioprocessing (CBP), the temperature of the microbial conversion must be adapted to the temperature enabling efficient polyester hydrolysis (Thomsen et al. 2022). One promising approach could be to engineer thermophilic strains for efficient growth on plastic monomers.

Several thermophilic bacteria have been described for their potential in industrial biotechnology. *Geobacillus* strains are of special interest (Hussein et al. 2015; Khaswal et al. 2022; Liu et al. 2024). They are Gram-positive, spore-forming bacteria known for their ability to thrive at high temperatures (optimal growth between 45 and 70 °C). Moreover, they feature a versatile catabolism and rapid growth rates, making them promising host strains in second-generation (lignocellulosic) or plastic waste biorefineries for biofuel or chemical production (Hussein et al. 2015).

In this work, we established robust growth of *Geobacillus thermoleovorans* on the PBAT monomers adipic acid (AA) and 1,4-butanediol (BDO) through adaptive laboratory evolution (ALE). To unravel the underlying catabolic pathways, the genomes of the evolved strains were sequenced and transcriptomic analyses were performed. Several candidates for enzymes involved in the catabolic reactions were identified. These enzymes indicated oxidative metabolism of BDO as well as metabolization of AA by CoA activation followed by β-oxidation.

The evolved strains were co-cultivated with PBAT as the sole carbon source in combination with supplemented HiC to primarily demonstrate simultaneous enzymatic PBAT hydrolysis and microbial monomer utilization as a basis for envisioned plastic upcycling approaches. Indeed, the cultivation resulted in the release and consumption of AA and BDO as well as growth of the *G. thermoleovorans* strains. Especially considering the possibility of purification of the remaining terephthalate (TA) by acid precipitation (Ismail et al. 2024), this demonstrates the potential of these strains for the development of microbial upcycling approaches.

## Experimental procedures

### Chemicals and microorganisms

Chemicals used in this study were purchased from Sigma-Aldrich (St. Louis, MO, USA), Carl Roth (Karlsruhe, Germany), or Merck (Darmstadt, Germany) unless stated otherwise. PBAT was obtained as beads from Bio-Mi sustainable solutions (Matulji, Croatia) and cryomilled by filling grinding jars with approximately 2 g of PBAT beads and a grinding ball (Ø 20 mm), 2–3 min of cooling in liquid nitrogen and milling at 20 Hz for 2 min with the MM 400 Mixer Mill (Retsch GmbH, Haan, Germany). The utilized strains were either purchased from Leibniz-Institut DSMZ-Deutsche Sammlung von Mikroorganismen und Zellkulturen (Braunschweig, Germany) or created via evolutionary engineering and are listed in Table 1.

**Table 1:** Strains utilized in this work and their relevant characteristics and references

Strain	Relevant characteristics	Reference
*G. stearothermophilus*	Type strain	DSM 458
*G. thermodenitrificans*	Type strain	DSM 465
*Parageobacillus thermoglucosidasius*	Type strain	DSM 2542
*G. thermoleovorans*	Type strain	DSM 5366
*G. thermoleovorans* BDO	*G. thermoleovorans* evolved on BDO	This work (MiKat#2649)
*G. thermoleovorans* AA	*G. thermoleovorans* evolved on AA	This work (MiKat#2648)
*G. thermoleovorans* AB	*G. thermoleovorans* AA evolved on BDO	This work (MiKat#3775)
*G. thermoleovorans* AA15	*G. thermoleovorans* AA evolved on a reduced concentration of AA	This work (MiKat#3264)

### Media and culture conditions

All cultivations were performed at 60 °C, 200 revolutions per minute (rpm) with an amplitude of 50 mm in an Infors HT Minitron incubator shaker (Infors AG, Bottmingen, Switzerland) or an ISF1-X Climo-Shaker (Kuhner shaker, Birsfelden, Switzerland). Depending on the desired culture volume and the planned duration of cultivation, the culture vessels used were either shake flasks (100, 250, or 500 mL) sealed with a metal cap and lab tape to reduce evaporation or 240-mL amber screw cap “Boston” bottles (Sigma-Aldrich, SKU #23235) with a screw cap with a central hole for a PTFE septum (Sigma-Aldrich, SKU #27022), which were tightly sealed to prevent evaporation completely. The filling volume was 10% of the total volume in the shake flasks and 10 mL in the Boston bottles.

The utilized media were LB medium containing 10 g L^−1^ peptone, 5 g L^−1^ sodium chloride, and 5 g L^−1^ yeast extract and mineral salts medium (MSM) adapted from Hartmans et al. (1989). The MSM contained either 20 mM glucose and a phosphate buffer capacity of 22.3 mM K_2_HPO_4_ and 13.6 mM NaH_2_PO_4_ or the PBAT monomers in the indicated concentrations and a threefold increased phosphate buffer capacity of 66.9 mM K_2_HPO_4_ and 40.8 mM NaH_2_PO_4_ or cryomilled PBAT in combination with 5 µM HiC and also threefold increased buffer capacity. Prior to utilization, the PBAT powder was sterilized with 70% (v/v) ethanol.

10 mL LB medium were inoculated with cells from a glycerol stock or with grown cells from an LB agar plate as initial pre-culture. After incubation overnight, the LB-culture was used to inoculate MSM with 20 mM glucose as a second pre-culture by adding 10% (v/v). A final pre-culture in the main culture medium was inoculated with 10% (v/v) from the glucose culture. The main culture in turn was inoculated with the final pre-culture to a starting optical density measured at 600 nm (OD_600_) of 0.1 unless stated otherwise.

For ALE experiments, the starting OD_600_ of each ALE culture was set to 0.05 and as soon as an OD_600_ above 0.5 was measured, a fresh culture was inoculated as a new ALE step. For isolation of single strains after an ALE, the final cultures were streaked on LB agar plates. These were incubated at 60 °C in an anaerobic jar (Anaerocult, 2.5-L volume, Merck KGaA, Darmstadt, Germany) with a few milliliters of water at the bottom to avoid drying out of the agar plates. Moreover, the jar was opened on a regular basis to ensure aerobic conditions.

### Bioreactor cultivations

*G. thermoleovorans* strains were cultivated in a 350 mL DASbox® mini-bioreactor system (Eppendorf SE, Hamburg, Germany) under the control of the DASware control software, using a working volume of 100 mL. The processes were carried out at 60 °C, 300 rpm agitation, and 1 vvm humidified headspace gassing. Oxygen availability was regulated via a dissolved oxygen (DO) cascade with a setpoint of 30%. When the DO deviated from the set value by 0–40%, the oxygen concentration in the headspace was enhanced gradually from 21 to 100%. Additionally, the stirring rate was raised from 300 to 1200 rpm, whenever the deviations exceeded 40%.

For continuous cultivations, the feed rate was adjusted to the indicated dilution rate. To maintain the working volume of 100 mL, a mechanical drain was applied. To this end, dip tubes were positioned at the liquid surface height and connected to external peristaltic pumps applying the waste flow (≥ threefold feed-flow rate). After at least five residence times, samples for RNA sequencing were collected. The pH was continuously monitored but not actively controlled.

For batch cultivations using cryomilled PBAT (25 g L^−1^) in combination with HiC (5 µM) as substrate, the pH was set to 7 via pH control with automatic base (2 M NaOH) and acid (2 M H₂SO₄) addition. Inoculation with the pre-culture and HiC addition, as well as the addition of various medium components for pulse experiments, were performed via a septum using a needle and syringe.

### Analytical methods

The growth of cultures was evaluated by measuring the OD_600_ using an Ultrospec 10 photometer (Biochrom, Cambridge, UK).

The concentrations of the different PBAT monomers as well as glucose were measured with a 1260 Infinity II HPLC system (Agilent, Santa Clara, CA, USA). Prior to HPLC analysis, samples were centrifuged for 3 min at 17,000 × *g*. The supernatants were subsequently filtered through an AcroPrep™ 96-well filter plate (Pall Corporation, Port Washington, NY, USA). Additionally, the samples were diluted tenfold for the TA measurement to ensure compatibility with the detection range. AA, BDO and glucose concentrations were all measured utilizing the column Metab-AAC (300 × 7.8 mm, ISERA; P.N.: A1BF-A1AA0N) combined with a Guard Cartridge Holder (ISERA, P.N.: AA13-000005) and Guard Column (10 × 7.8 mm, ISERA, P.N.: A1BF-A1AG0N). Chromatographic separation was performed using 5 mM H₂SO₄ as the mobile phase at a column temperature of 40 °C and a flow rate of 0.6 mL min^−1^. Detection of the components was conducted with a 260 Infinity II Refractive Index Detector (Agilent, Santa Clara, California, USA). Measurement of the TA concentration was carried out utilizing the ISAspher 100–5 C18 BDS column (Isera, Düren, Germany) with a gradient of 0.1% (v/v) trifluoroacetic acid (Sigma-Aldrich, Munich, Germany) and acetonitrile (Th. Geyer, Renningen, Germany) at a flow rate of 0.8 mL min^−1^ and a column temperature of 40 °C. TA was UV-detected at a wavelength of 280 nm with the 1260 DAD WR detector (Agilent, Santa Clara, CA, USA). To quantify all compounds of interest, standard solutions with known concentrations were measured and used to generate calibration curves. For BDO, AA, and TA, the lowest standard concentration of 1 mM was reliably quantified, indicating a lower detection limit of 1 mM for all three compounds.

### Whole genome sequencing (WGS)

For WGS, genomic DNA was isolated using the Monarch® Genomic DNA Purification Kit (New England Biolabs, Ipswich, MA, USA) following the manufacturer’s instructions. The isolated DNA from all *G. thermoleovorans* strains was subjected to short-read sequencing using the in-house MiSeq instrument (Illumina, San Diego, CA, USA) according to the procedure described by Lechtenberg et al. (2024). Additionally, the unevolved strain and the strain *G. thermoleovorans* AB were subjected to long-read sequencing (Oxford Nanopore Technologies) performed by Eurofins Genomics (Ebersberg, Germany). The initial quality of the raw reads was assessed using FastQC (v0.11.9) (https://www.bioinformatics.babraham.ac.uk/projects/fastqc) for the short reads and LongQC (v1.2.1) for the long reads (Fukasawa et al. 2020). Following quality assessment, reads were trimmed to remove adapters and low-quality bases using Trimmomatic (v0.39) for Illumina data (Bolger et al. 2014) and Porechop (v0.2.4) for Nanopore data (https://github.com/rrwick/Porechop), both with default parameters. A de novo genome assembly of the reference genome from the unevolved *G. thermoleovorans* strain was completed using a hybrid strategy that incorporated both Illumina short reads and Nanopore long reads. First, a preliminary assembly was constructed from the trimmed long reads using Flye (v2.9) (Kolmogorov et al. 2019). The contigs from this initial assembly were then supplied as a trusted reference to SPAdes (v4.0.0), which performed a hybrid assembly using both the long and short reads (Bankevich et al. 2012). The draft genome from the hybrid assembly was polished to correct base-level errors and close remaining gaps. This was accomplished using a combination of Pilon (v1.24) (Walker et al. 2014) and ABySS-Sealer (v2.1.0) (Paulino et al. 2015). The final, polished genome was then annotated to identify coding sequences and other genomic features using Prokka (v14.5) (Seemann 2014). To improve the accuracy of gene prediction and annotation, RNA sequencing reads were additionally incorporated into the analysis.

For SNP detection and transcriptomic analysis, the de novo–assembled genome of the unevolved *G. thermoleovorans* strain served as the reference. For the genomes of the evolved strains sequenced by MiSeq sequencing only, the resulting read data (FASTQ files) were processed with the CLC Genomics Workbench software (Qiagen Aarhus A/S) for base quality filtering and read trimming. With the resulting mappings, gene coverage analysis and quality-based SNP as well as structural variant detection were performed also with the CLC Genomics Workbench software (Qiagen Aarhus A/S). For the *G.thermoleovorans* AB sample, pre-processed long reads were aligned to the *G. thermoleovorans* reference genome assembly using Minimap2 v2.24 with the preset parameters for Oxford Nanopore data (-ax map-ont) (Li 2018). The resulting alignments were converted to the binary format (BAM), sorted by coordinate, and indexed using Samtools v1.15 (Danecek et al. 2021). SNPs were identified from the sorted BAM file using Medaka v1.7.2 (https://github.com/nanoporetech/medaka). The functional effects of the identified variants were predicted using SnpEff v5.1 (Cingolani et al. 2012). A custom SnpEff database was constructed for the *G. thermoleovorans* reference genome using the genome sequence and its corresponding GFF3 annotation file. The variant annotation was then performed using this custom database, and the final output was generated as an annotated VCF file.

Sequencing data have been deposited in the European Nucleotide Archive under BioProject number PRJEB104702. Additionally, all raw data, computational workflows and results are accessible as an Annotated Research Context FAIR Digital Object under: 10.60534/y7jxb-5r565 (Weil et al. 2023).

### RNA sequencing

Samples for RNA sequencing were taken from chemostat cultivations after at least five residence times. The total RNA was isolated using the Monarch® Total RNA Miniprep Kit following the instructions of the manufacturer (New England Biolabs, Ipswich, MA, USA). The concentration as well as integrity of the RNA was measured and verified with the 4200 TapeStation System and the corresponding RNA ScreenTape Analysis and RNA ScreenTape Sample Buffer (all from Agilent, Santa Clara, California, USA). Depletion of rRNA, library preparation, and sequencing were conducted by Genewiz Azenta Life Sciences (Chelmsford, Massachusetts, USA).

Analysis of the transcriptome data received from Genewiz was performed with the CLC Genomics Workbench 20.0.4 software (Qiagen, Hilden, Germany). As a first step, low-quality reads (0.05), ambiguous nucleotides and adapter sequences were trimmed as part of a quality control. The resulting reads were mapped to the *G. thermoleorovorans* reference genome and transcripts-per-million (TPM) values were calculated using the RNA-seq analysis tool of CLC Genomics Workbench (read alignment parameters: mismatch cost, 2; insertion cost, 3; deletion cost, 3; length fraction, 0.8; similarity fraction, 0.8; strand specificity, both; maximum number of hits for a read, 10). Subsequently, statistical analysis of differential gene expression between strains and carbon sources tested was carried out using default settings of the CLC Genomics Workbench 20.0.4 software (Qiagen, Hilden, Germany) (Robinson and Smyth 2008). The FDR-adjusted *p*-value (Padj) was used to correct false positives among the set of statistically significant results in multiple hypothesis testing (Benjamini and Hochberg 1995).

## Results and discussion

### Improving growth of *Geobacillus* on 1,4-butanediol and adipate

**Fig. 1:**
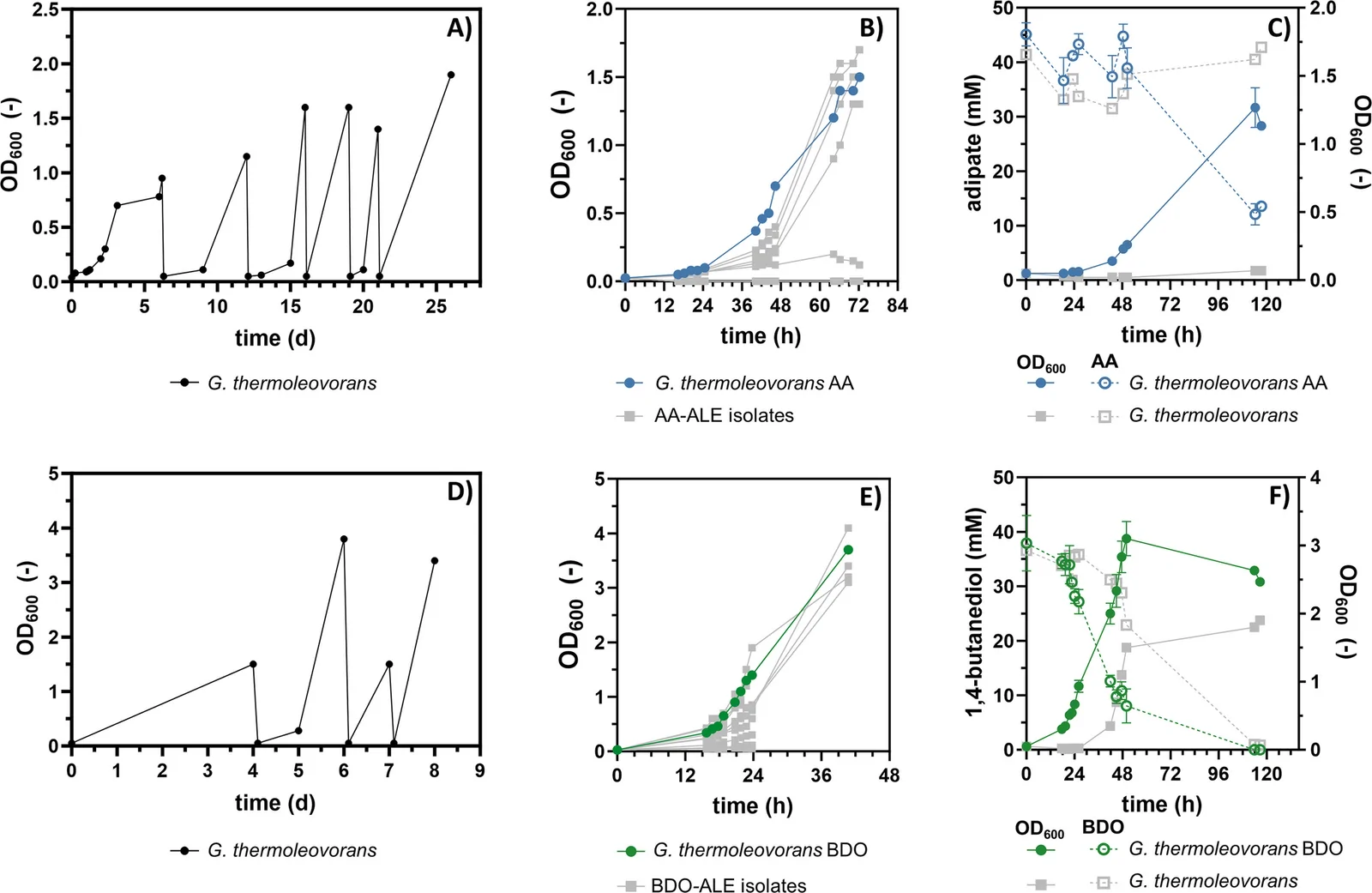
Adaptive laboratory evolution (ALE) of* G. thermoleovorans *on adipate (AA) and 1,4-butanediol (BDO). **A** and **D** Sequential batch cultivation of *G. thermoleovorans* in 10 mL mineral salt medium (MSM), with 30 mM AA (**A**) or 45 mM BDO (**D**) as the sole carbon source in Boston bottles. **B** and** E** Cultivation of multiple (≥ 6) single clones resulting from the final culture of the ALE on 30 mM AA (**B**) and 45 mM BDO (**E**) to select evolved strains with improved growth. The growth curves of the selected strains are shown in blue (AA) or green (BDO), and the growth curves of all remaining strains are shown in grey. **C** and **F** OD_600_ and substrate concentrations measured at different time points during the cultivation of the selected evolved strain on 30 mM AA (**C**) or 45 mM BDO (**F**) in shake flasks. The substrate concentrations are shown as dashed lines with open symbols. Error bars in **C** and **F** are derived from three replicates and indicate the standard error of the mean (SEM). Unevolved wild type, grey (*n* = 1)

The growth of three different *Geobacillus* strains, *G. thermodenitrificans* (DSM 465), *G. stearothermophilus* (DSM 458), and *G. thermoleovorans* (DSM 5366), as well as *Parageobacillus thermoglucosidasius* (DSM 2542), was tested on the monomers of the biodegradable plastic PBAT at 60 °C. Slow growth was exclusively detected during cultivation of *G. thermoleovorans* on BDO and AA, and only after an extended cultivation time of approximately 48 and 60 h, respectively. None of the tested strains grew on TA. To enhance growth performances of this strain, two separate ALE campaigns were conducted on AA (Fig. 1A) and BDO (Fig. 1D) as sole carbon sources. Single clones were isolated from the final ALE cultures by streaking on LB agar plates and subsequently tested for growth on their respective ALE substrate (Fig. 1B and E) after intermediate cultivation in non-selective media (LB and MSM with glucose). For each substrate, clones exhibiting improved growth compared to the unevolved wild type were obtained, and the strain with the best combination of growth rate and final OD_600_ was selected for further investigation. The two selected evolved strains, denoted *G. thermoleovorans* AA and *G. thermoleovorans* BDO, grew with a rate of 0.054 ± 0.004 h^−1^ on AA (Fig. 1C) and 0.13 ± 0.010 h^−1^ on BDO (Fig. 1F), respectively. Complete metabolization of BDO by the evolved strain was achieved, whereas no decrease in AA below 15 mM was observed even after prolonged incubation of the culture in the stationary phase. This could indicate that the uptake of AA is mediated by a transporter with a high *K*_m_ value for this substrate (Diallinas 2014). However, other reasons limiting growth on AA at concentrations below 15 mM cannot be excluded, and further investigation is needed to fully elucidate the underlying effects. Moreover, it should be noted that the shorter lag phases observed during some of the sequential ALE cultures (Fig. 1A and D) compared to the shake flask cultivations of the selected evolved strains (Fig. 1C and F) are likely due to differences in the physiological state of the inoculum. During ALE, cultures were occasionally used to inoculate subsequent ALE steps while still in the exponential growth phase, whereas the shake flask cultivations were inoculated with cultures grown on their respective substrate after reaching stationary phase.

### Unraveling the pathways for adipate and 1,4-butanediol catabolism through genomic and transcriptomic analysis

**Fig. 2:**
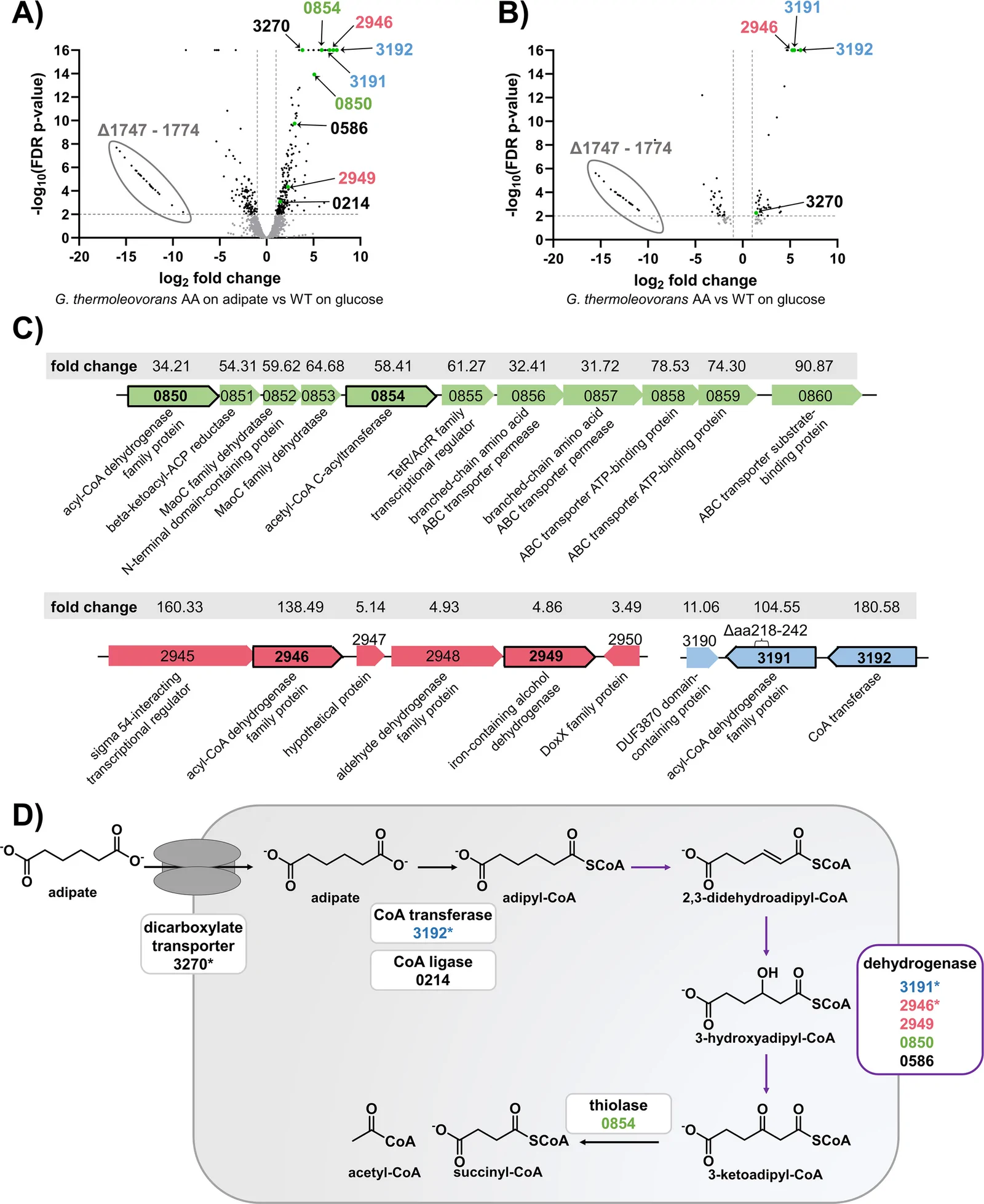
The proposed AA metabolism in evolved *G.thermoleovorans* AA. The numbers refer to the gene-IDs in the reference genome available in the European Nucleotide Archive under BioProject number PRJEB104702. The gene-IDs are abbreviated for readability (prefix “Gth_00” omitted).** A** and** B** Volcano plots of differentially expressed genes (DEG). Insignificant hits (FDR *p*-value ≥ 0.01 or |log_2_ fold change|< 1) are displayed in grey. The thresholds are displayed as grey lines. DEG hypothesized to be involved in AA metabolism are displayed in green, and the corresponding Gth-number is indicated in the color corresponding to the genomic contexts shown in (**C**). A full list of significantly upregulated and downregulated genes is shown in Table S3 to Table S6. Differential gene expression resulting from the comparison of *G. thermoleovorans* AA grown on 30 mM AA (**A**) or 30 mM glucose (**B**) against the unevolved *G. thermoleovorans* grown on 30 mM glucose. **C** Genomic context of genes potentially involved in AA metabolism that are organized in clusters upregulated during growth on AA. Genes with black frames are part of the proposed AA metabolism shown in (**D**). The linear fold change of the respective genes is shown above them against a grey background. Δaa indicates deleted amino acids. **D** The proposed pathway for AA metabolization. Colors of the enzymes correspond to the genomic contexts shown in (**C**). Asterisks indicate upregulation of the encoding gene during cultivation on AA and glucose

**Fig. 3:**
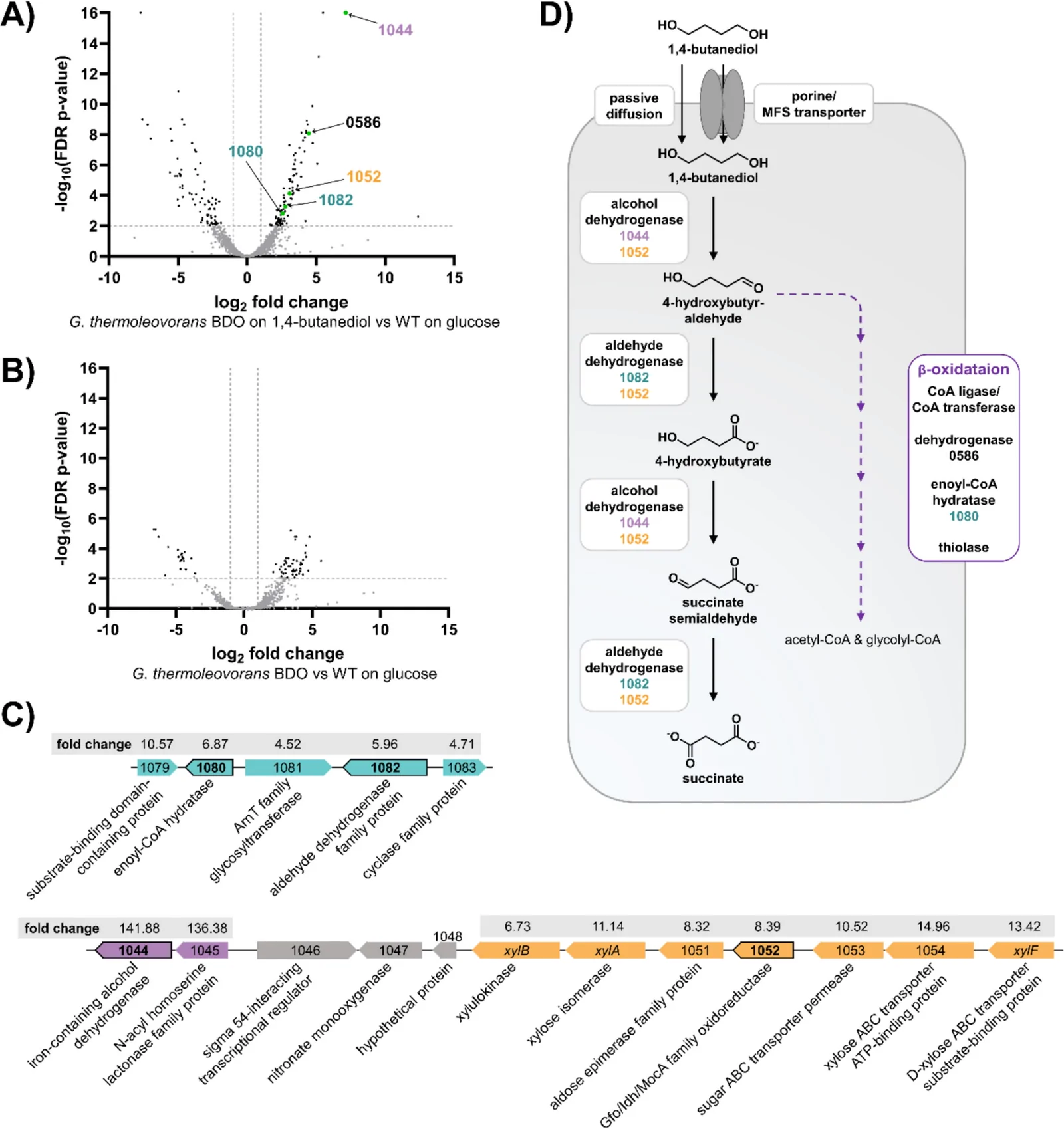
The proposed BDO metabolism in evolved *G. thermoleovorans* BDO. The numbers refer to the gene-IDs in the reference genome available in the European Nucleotide Archive under BioProject number PRJEB104702. The gene-IDs are abbreviated for readability (prefix “Gth_00” omitted).** A** and** B** Volcano plots of DEG. Insignificant hits (FDR *p*-value ≥ 0.01 or |log_2_ fold change|< 1) are displayed in grey. The thresholds are displayed as grey lines. DEG hypothesized to be involved in BDO metabolism are displayed in green, and the corresponding Gth-number is indicated in the color corresponding to the genomic contexts shown in (**C**). A full list of significantly upregulated and downregulated genes is shown in Table S7 to Table S10. Differential gene expression resulting from comparison of *G. thermoleovorans* BDO grown on 45 mM BDO (**A**) or 30 mM glucose (**B**) against the unevolved *G. thermoleovorans* grown on 30 mM glucose. **C** Genomic context of genes potentially involved in BDO metabolism that are organized in clusters upregulated during growth on BDO. Colored genes represent DEG; grey genes were not identified in the transcriptomics. Genes with black frames are involved in the proposed BDO metabolism shown in (**D**). The linear fold change of the respective genes is shown above them against the grey background.** D** The proposed pathway for BDO metabolization. The colors of the enzymes correspond to the genomic contexts shown in (**C**)

After evolved clones with enhanced growth on AA and BDO were successfully obtained, we investigated the genetic basis underlying these phenotypes. Therefore, the genomes of the best-performing strains were sequenced. Moreover, a high-quality reference genome of the unevolved parent strain was generated utilizing a hybrid de novo assembly approach combining Illumina short reads and Oxford Nanopore long reads. The resulting genome was annotated with Prokka (Seemann 2014). To enhance the accuracy of gene prediction and annotation, RNA sequencing data obtained from the transcriptomic analysis described below were also integrated into the annotation. The genome is publicly available in the European Nucleotide Archive under BioProject number PRJEB104702. All mutations that were identified in the evolved strains on AA and BDO are listed in Table S1 and Table S2 in the supplementary material. Additionally, RNA sequencing was performed for the AA and BDO strains to identify differentially expressed genes (DEG) and suggest enzymes and pathways involved in AA or BDO metabolism (Figs. 2 and 3).

Through genome sequencing, several interesting mutations were identified in *G. thermoleovorans* AA that could potentially be related to the improved growth on AA. Particularly striking is a deletion of a large genomic segment of over 15.6 kb. The associated segment comprises 27 genes, most of which were annotated as hypothetical proteins, but also as a putative recombinase family protein, an ATPase, two helix-turn-helix transcriptional regulators, and more. The deletion of this large region indicates that the included genes did not provide any advantage for the cells under ALE conditions, and it may therefore have arisen and been passed on simply by coincidence. However, its absence could also display a growth advantage on AA, especially with regard to the deletion of the two genes coding for regulators.

Further, the *pnp* gene was altered in both the AA-evolved and BDO-evolved strains. Therefore, it is likely the result of an adaptation to more general features such as growth under laboratory conditions and not specific to growth on AA or BDO. This gene encodes a polyribonucleotide nucleotidyltransferase, which is involved in mRNA degradation, and may play a role in a stress-response mechanism (Vargas-Blanco and Shell 2020). Mutations enhancing growth under general laboratory conditions rather than on the specific substrate appear to be a recurring trend across the ALE campaigns performed in this study, suggesting that *G. thermoleovorans* is not well adapted to the overall laboratory environment.

In addition, a 75-bp in-frame deletion in the coding region of Gth_003191 annotated as acyl-CoA dehydrogenase was identified, causing a deletion of 25 from a total of 399 amino acids. As the deletion did not lead to a frameshift, a gain-of-function cannot be excluded. However, Alphafold 3 predicted a complete alteration of the protein structure, therefore pointing toward a loss-of-function mutation (Abramson et al. 2024).

The other identified mutations are mostly located in intergenic regions (IGR) or they are silent mutations within coding regions. While the mutations in the IGR may affect regulatory elements, thereby influencing gene expression and potentially contributing to the observed phenotype, such regulatory sequences remain poorly defined in *Geobacillus* species and are difficult to predict (Gilman et al. 2019). For this reason, no definitive conclusions were made regarding the relevance of these mutations on the regulatory level. The silent mutations within coding regions in turn might influence expression levels by altering codons to less or more favorable ones, thereby influencing translation efficiency, or by altering transcription factor binding sites that can occur in the 5’-end of the gene. However, even when considering these potential effects, no connection to AA metabolism is apparent based on the annotations of the mutated genes and their immediate genomic context. Therefore, their potential role in AA metabolism cannot be elucidated solely based on the genome sequencing data. For this reason, transcriptomic analysis was performed by RNA sequencing (Fig. 2A and B).

Transcriptomics offers the advantage of detailed analysis of genes involved in adaptive responses under a specific condition. However, gene expression is strongly dependent on various environmental conditions, such as the concentration of the substrate(s) and other medium components, and especially the growth rate (Mundinger et al. 2019; Tai et al. 2005). To minimize the influence of the growth rate, chemostat cultivations were carried out (Fig. S1) (Boer et al. 2007). The evolved strains were each cultivated at a specific dilution rate on their respective substrate and on glucose. The dilution rates were 0.15 h^−1^ for *G. thermoleovorans* BDO and 0.04 h^−1^ for *G. thermoleovorans* AA. In addition, the unevolved strain was also cultivated on glucose at both dilution rates. By comparing DEG for the strains grown at the same dilution rate, the influence of different growth rates on gene expression can be abolished. Comparison of the DEG in the evolved strains on glucose with the non-evolved strain on glucose allows identification of genes permanently differently expressed due to mutations that occurred during the ALE (Figs. 2B and 3B). Additional comparison with the DEG in the evolved strain when cultivated on AA/BDO enables the identification of genes temporarily differentially expressed as an adaptation to the different carbon source (Figs. 2A and 3A). All identified DEG for *G. thermoleovorans* AA are listed in Tables S3 to S6 in the supplements.

Based on the transcriptomics data, the putative metabolic pathway for the metabolization of AA shown in Fig. 2D is proposed. First, AA is transported into the cytoplasm via a dicarboxylate transporter encoded by Gth_003270. This gene is upregulated during growth of the evolved strain on both glucose and AA, suggesting that a mutation occurred during ALE, which causes its constitutive expression. However, no mutation potentially responsible for this effect could be identified via genome sequencing. Moreover, higher expression levels observed during growth on AA indicate an additional, substrate-specific upregulation.

In the cytoplasm, one of the carboxyl groups of AA is CoA activated. This can be catalyzed by a CoA ligase using ATP or by a CoA transferase using another CoA-activated substrate such as acetyl-CoA (Hackmann 2022; Wang et al. 2018). During cultivation on AA, genes coding for both enzyme types were upregulated. However, the expression of the Gth_003192 encoding the CoA transferase was considerably higher than the expression of Gth_000214 encoding the CoA ligase. Additionally, enhanced expression of Gth_003192 was also measured during cultivation of the evolved strain on glucose, suggesting a constitutive expression in the evolved strain. This might be caused by the abovementioned deletion in the neighboring gene Gth_003191 encoding an acyl-CoA dehydrogenase. Surprisingly, the mutated gene Gth_003191 also showed strong upregulation. A potential cause might lie in an increased mRNA stability due to the altered sequence, resulting in a higher accumulation of transcripts (Belasco and Biggins 1988). Assuming the mutation led to the destruction of the tertiary structure of the encoded enzyme, as indicated by Alphafold 3 prediction, the overexpression of this gene would offer no apparent advantage to the evolved strain. In this case, it is rather a side effect of the upregulation of the downstream gene enabling CoA activation of AA. Alternatively, the structure prediction might also be incorrect, which appears plausible given the potential role of the encoded enzyme in the proposed β-oxidative catabolic AA pathway (Fig. 2D). However, further investigation is necessary to fully clarify the precise effect of this deletion.

The activated adipyl-CoA is expected to be further metabolized via β-oxidation. Various enzyme candidates potentially involved in this were identified via transcriptomics. These include Gth_002946 annotated as an acyl-CoA dehydrogenase family protein. This gene is part of a gene cluster, which was upregulated in the evolved strain on AA and glucose (Fig. 2C). It comprises five genes encoding an iron-containing dehydrogenase, Gth_002949, which could also be involved in the oxidation steps, a sigma-54-interacting transcriptional regulator, an aldehyde dehydrogenase, and a hypothetical protein. Although the cluster was strongly expressed on AA and glucose, indicating its constitutive expression, genome sequencing did not reveal any mutations in the immediate vicinity of this gene cluster. Its strong expression might therefore be caused by a more distal regulatory mutation. The upregulation on AA might additionally be caused by improved uptake, which enabled AA-mediated activation of native transcriptional regulators. Furthermore, two more genes, also annotated as acyl-CoA dehydrogenase family proteins, Gth_00586 and Gth_00850, were upregulated during growth on AA and are potentially catalyzing the β-oxidation steps in AA degradation.

Once the adipyl-CoA has been oxidized to 3-ketoadipyl-CoA, cleavage into acetyl-CoA and succinyl-CoA is catalyzed by a thiolase. A potential candidate is Gth_000854, which is part of the gene cluster that also harbors Gth_00850. The resulting acetyl-CoA and succinyl-CoA are further metabolized via the central carbon metabolism.

Regardless of the substrate, the gene cluster from Gth_001747 to Gth_001774 was identified as strongly downregulated in the evolved strain, consistent with its deletion. Conversely, this means that the gene cluster was expressed in the wild type. This supports the hypothesis that the deletion saves resources and is thus advantageous under the chosen cultivation settings.

All mutations identified in the genome of the BDO-evolved strain are listed in Table S2. Surprisingly, WGS did not reveal any mutations that can be directly linked to BDO metabolism, despite the stable improvement in growth on BDO. One possible explanation is that the observed phenotype partly results from more general adaptations to laboratory conditions rather than from mutations specifically enhancing BDO metabolism. One example of such a mutation is the SNV in *pnp*, as already discussed above. In addition, global regulatory effects, which cannot be revealed solely from WGS data, may contribute to the observed phenotype. Therefore, transcriptomic analysis was also conducted for this strain to identify phenotype-associated changes in gene expression and thereby gain further insights into BDO metabolism. The identified DEG are listed in Tables S7 to S10 in the supplements. Several of them could potentially be assigned roles in BDO metabolism as proposed in Fig. 3D. Interestingly, all of them were only upregulated on BDO and not on glucose. This contradicts the observation that the evolved strain grows significantly faster than the wild type, indicating a permanent change in the expression of relevant genes or a structural change in an enzyme that subsequently takes over a required function. A possible cause could lie in improved BDO uptake, resulting in activation of native transcriptional regulators by BDO functioning as an inducer. However, no potential transporter was identified by WGS or transcriptomic analysis, suggesting uptake via passive diffusion or a constitutively expressed transporter. In the cytoplasm, one of the alcohol groups is initially oxidized by an alcohol dehydrogenase. Two potential candidates were identified for this: Gth_001044, which is highly expressed, making its involvement in BDO metabolism likely, and Gth_001052. The resulting aldehyde group is further oxidized by an aldehyde dehydrogenase potentially encoded by Gth_001082 or Gth_001052. For further degradation of 4-hydroxybutyrate, two different pathways are possible, which were also proposed for *P. putida* and *P. taiwanensis* before (Li et al. 2020; Op de Hipt et al. 2025): 1) CoA activation of the carboxyl group and degradation via β-oxidation resulting in acetyl-CoA and glycolyl-CoA or 2) oxidation of the second alcohol group via an aldehyde intermediate to a carboxyl group resulting in succinate.

For the first route, either a CoA ligase or CoA transferase must be expressed to enable CoA activation of 4-hydroxybutyrate. However, none of the two enzymes could be identified in the DEG. In addition, no thiolase required for the final conversion of 4-hydroxy-3-keto-butyryl-CoA to acetyl-CoA and glycolyl-CoA could be identified either. The only gene that might be involved in this pathway, which is significantly upregulated, is Gth_000586 encoding an acyl-CoA dehydrogenase family protein. This makes it more likely that BDO degradation occurs via the second pathway.

In this route, the enzyme candidates identified for the oxidation of the first alcohol group theoretically could also catalyze the oxidation of the second alcohol. However, previous studies on *Pseudomonas* strains engineered for medium-chain-length diol metabolization have shown that the second oxidation step is typically carried out by a distinct enzyme, better suited to accommodate the charged carboxylate group formed during the initial oxidation (Ackermann et al. 2024; Li et al. 2020; Op de Hipt et al. 2025).

### Cultivation of evolved *G. thermoleovorans* on PBAT as sole carbon source

**Fig. 4:**
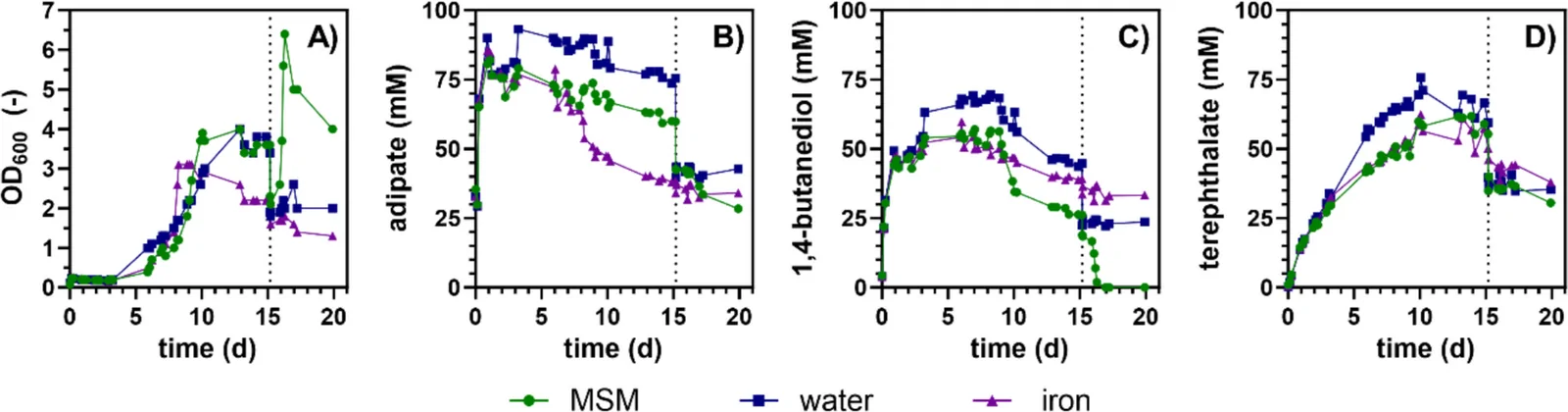
One-pot hydrolysis of PBAT by HiC and growth of evolved *G. thermoleovorans* on the released AA and BDO. The process was conducted in DASbox® mini-bioreactors filled with 100 mL MSM with 25 g L^−1^ PBAT powder and 5 µM HiC. The starting OD_600_ of each strain was 0.05. Samples were taken daily during the whole process, and the OD_600_ (**A**), AA (**B**), BDO (**C**), and TA concentration (**D**) were measured. On day 15, after growth stagnated in all reactors, a different pulse was added to each reactor. Symbol shapes and colors represent the bioreactors with their designated pulse: MSM (green circle), water (blue square), 30 mg L^−1^ iron (purple triangle)

The intended application of the engineered strains lies in the field of plastic upcycling. An interesting material with increasing relevance as a sustainable alternative especially for single-use plastics is the polyester PBAT (Itabana et al. 2024), which can be enzymatically hydrolyzed at 60 °C using various enzymes, including the commercially available cutinase HiC (Perz et al. 2016). To evaluate the potential of the evolved strains for application in plastic upcycling CBP, a bioreactor co-cultivation of *G. thermoleovorans* AA and BDO was performed using PBAT as the sole carbon source in combination with HiC for enzymatic degradation of this polymer (Fig. 4).

Immediately after enzyme addition and inoculation, 33.5 ± 0.6 mM of AA was detected, probably due to leaching of residual unpolymerized AA from the PBAT production process. In subsequent experiments using PBAT from a different batch supplied by the same manufacturer, no initial AA was observed, indicating batch-to-batch variability. While differences between the three reactors in substrate concentrations and OD_600_ were observed, indicating variability between replicates and warranting a cautious interpretation of the data, general trends could still be identified. During the cultivation, an increase in OD_600_ and a decrease in AA and BDO concentrations were detected, demonstrating the applicability of the strains in combination with HiC for PBAT degradation. After 15 days, however, a stagnation in OD_600_ was observed in all reactors although AA and BDO monomers were still present. Different solutions were pulsed to each reactor to identify limiting factors. A water pulse was applied to dilute potentially critical concentrations of inhibitory components, and iron, as well as an MSM pulse, was added to identify potential micronutrient limitations. All pulses led to an immediate decrease in concentrations and OD_600_ due to dilution effects, but only the MSM pulse resulted in a subsequent increase in OD_600_. Simultaneously, a decrease in BDO and AA concentrations was observed. While BDO was fully metabolized, AA consumption remained incomplete and stopped at 28 mM. This incomplete AA consumption was also observed during cultivation of the AA-degrading strain in shake flasks (Fig. 1C), even after prolonged incubation beyond the stationary phase, suggesting that this limitation is strain-specific rather than caused by process parameters. The OD_600_ dropped upon prolonged incubation, which can likely be ascribed to morphological changes. Overall, this suggests that a medium component other than iron, such as ammonium or a trace element, was limiting.

In summary, this experiment highlights the potential of the evolved *G. thermoleovorans* strains for use in PBAT upcycling CBP. Furthermore, the improved growth after the addition of fresh MSM underlines that additional process optimization is required, particularly concerning the composition of the medium, to render such CBP as efficient as possible.

### Combination of adipate and 1,4-butanediol metabolism in one strain

Apart from the optimization of the medium and process conditions, further optimization of the evolved *G. thermoleovorans* strains is also conceivable to enhance the efficiency of potential CBP. The co-culture approach presented in this study (Fig. 4) offers distinct advantages, particularly in terms of flexibility, as it can enable selective monomer consumption depending on the chosen strains and allow adjustment of inoculum ratios to balance differences in substrate uptake kinetics. Nevertheless, the use of one strain co-metabolizing several plastic monomers can also be advantageous. As with classical diauxic growth, initial consumption of a preferred substrate can result in a higher cell density, which accelerates the volumetric rate of degradation of a second more difficult-to-metabolize substrate. In contrast, in mixed cultures, the duration of the process is solely determined by the slowest strain. In addition, the process complexity will be reduced by avoiding competition and co-operation dynamics between the different strains (Khedkar et al. 2024).

**Fig. 5:**
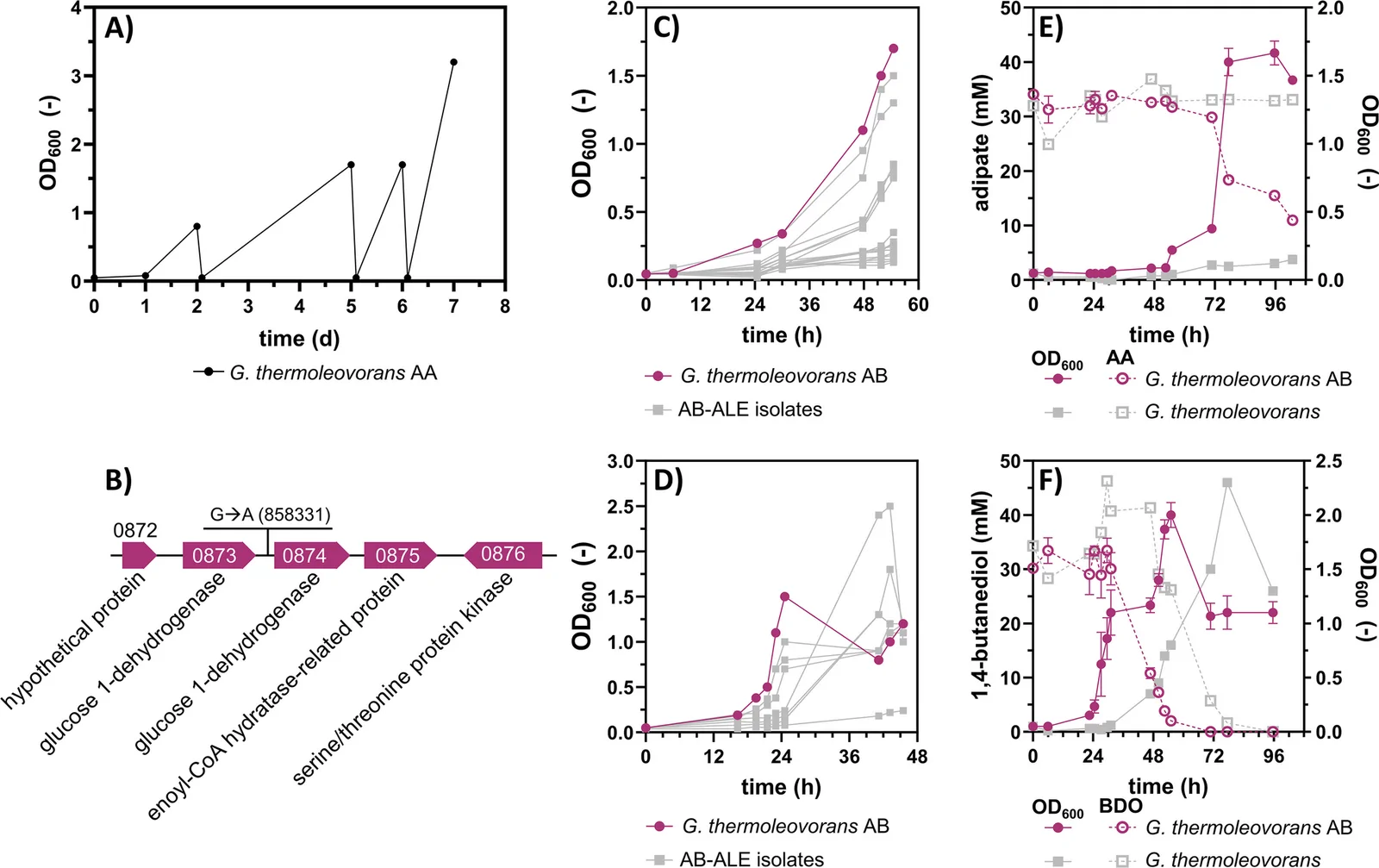
ALE of *G. thermoleovorans* AA on BDO.** A** Sequential batch cultivation of *G. thermoleovorans* AA in 10 mL MSM with 45 mM BDO as sole carbon source in Boston bottles. **C** and** D** Cultivation of multiple (≥ 6) single clones resulting from the final culture of the ALE on 30 mM AA (**C**) and 45 mM BDO (**D**) to select an evolved strain with improved growth on both substrates. The growth curves of the chosen strain are shown in purple and those of all the remaining strains are shown in grey. **E** and **F** OD_600_ and substrate concentrations measured at different time points during the cultivation of a selected evolved strain (purple) and the unevolved wild type strain (grey) on 30 mM AA (**E**) or 45 mM BDO (**F**) in shake flasks. The substrate concentrations are shown as dashed lines with open symbols. Error bars in (**E**) and (**F**) are derived from three replicates and indicate the SEM. Unevolved wild-type, grey (*n* = 1). **B** Genomic context of a putative causal mutation of *G. thermoleovorans* AB. The numbers refer to the gene-IDs in the reference genome available in the European Nucleotide Archive under BioProject number PRJEB104702. The gene-IDs are abbreviated for readability (prefix “Gth_00” omitted)

Therefore, an additional ALE on BDO was performed with *G. thermoleovorans* AA. During the ALE, an increase in OD_600_ was observed along with a decrease in cultivation times (Fig. 5A). Isolated clones were cultured on AA (Fig. 5C) and BDO (Fig. 5D) to select a strain that showed improved growth on both substrates, yielding *G. thermoleovorans* strain AB that exhibited the fastest growth on AA and BDO. Notably, growth was also improved compared to *G. thermoleovorans* BDO and *G. thermoleovorans* AA on the respective carbon source. The improved growth on AA could indicate that not only mutations enhancing growth on BDO occurred, but also mutations providing a general growth advantage under the given culture conditions may have emerged. Furthermore, crosstalk between the two metabolic pathways and shared enzymes capable of catalyzing reactions in both catabolic routes are conceivable. This is particularly plausible if AA and BDO are both degraded via CoA activation followed by β-oxidation. Due to such overlaps in the degradation pathways, it is possible that mutations selected during ALE on BDO, which enhance the efficiency of the shared pathway steps, could lead to improved growth on both substrates, as previously observed with *P. putida* on AA and 1,6-hexanediol (Ackermann et al. 2024).

To reveal the underlying genomic changes, WGS of *G. thermoleovorans* AB was performed and all identified mutations are listed in Table S11 in the supplementary material. Among these, one especially promising mutation, which is likely to influence growth on BDO positively, is a single-nucleotide variant (SNV) in the intergenic region between Gth_001082 and Gth_001083. These genes and other genes in the genomic context including Gth_001080 and Gth_001081 were also found to be expressed during growth of *G. thermoleovorans* BDO on BDO and Gth_001080 as well as Gth_001082 are part of the proposed BDO catabolic pathway (Fig. 3D). Gth_001082 encodes an aldehyde dehydrogenase that presumably catalyzes the oxidation of 4-hydroxybutyraldehyde to 4-hydroxybutyrate and potentially also of succinate semialdehyde to succinate, assuming BDO metabolism also occurs via the direct oxidation route. In the same genomic region, Gth_001080 encodes an enoyl-CoA hydratase that likely catalyzes the hydration step of the enoyl-CoA intermediate during the putative β-oxidation of AA and BDO. In accordance, enhanced expression of Gth_001080 potentially alleviates a bottleneck during the metabolization of both AA and BDO. The mutation is likely to result in an alteration of a regulatory DNA sequence thereby enabling enhanced expression of Gth_001082. Gth_001080 lies in the same orientation, suggesting that the expression of both genes is affected by the mutation, but the interspacing gene Gth_001081 lies in the opposite orientation.

Similarly, a mutation was identified in the IGR between Gth_000873 and Gth_000874, both encoding short-chain dehydrogenases/reductases (SDR) family NAD(P)-dependent oxidoreductases (Kavanagh et al. 2008). More specifically, they were annotated as glucose-1-dehydrogenase by Prokka as well as the PGAP annotation pipeline (Seemann 2014; Tatusova et al. 2016). However, SDR are known for a remarkably wide substrate spectrum and even minor alterations in the substrate-specificity-determining loops of the enzymes can result in an altered specificity (Kavanagh et al. 2008; Persson and Kallberg 2013; Qian et al. 2024Tanaka et al., 2005). Therefore, annotation of these enzymes as glucose-1-dehydrogenases may be misleading, and it is plausible that they also accept other polar substrates, such as BDO and/or intermediates of its oxidative degradation. In this case, the mutation is likely to result in higher expression of the neighboring genes and enhanced BDO degradation via oxidation by the encoded dehydrogenases. Moreover, the upstream gene Gth_000875 is annotated as enoyl-CoA hydratase-related protein and therefore might, similarly to the mutation described before, be involved in the hydration step of the β-oxidation. Both mutations combined indicate that this step is indeed rate-limiting in *G. thermoleovorans* AA and *G. thermoleovorans* BDO.

About 30 more mutations were identified in *G. thermoleovorans* AB, for which no obvious connection to either AA or BDO metabolism is apparent. However, an impact of these mutations on the regulatory and/or global level cannot be excluded, and it is possible that they also improve cellular fitness and growth under the given cultivation conditions.

**Fig. 6:**
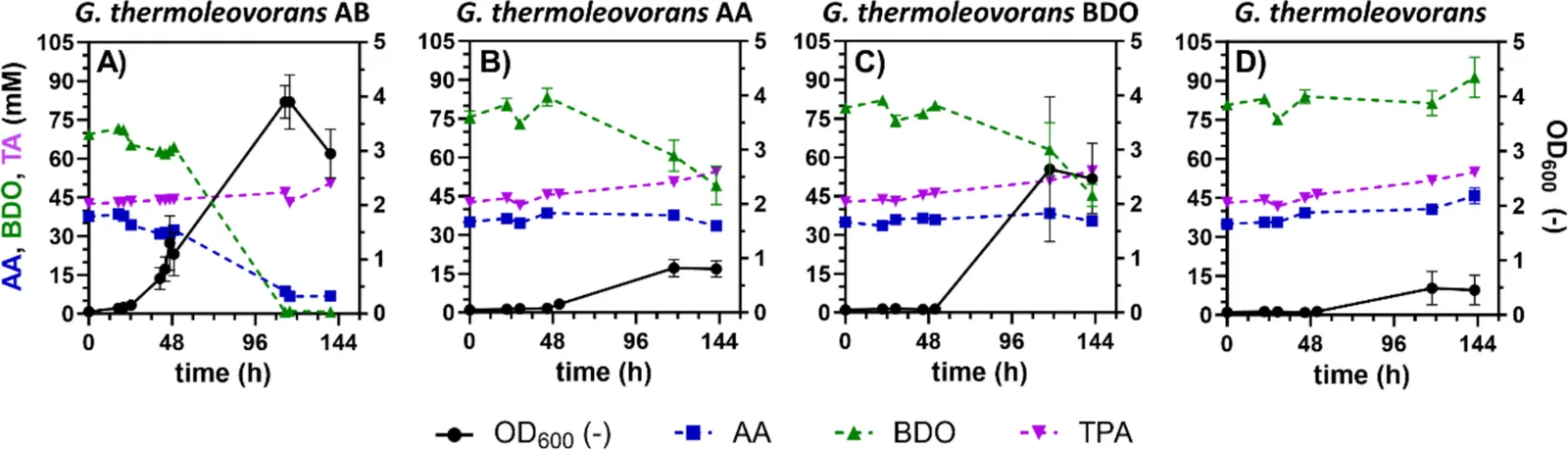
Cultivation of evolved *G. thermoleovorans* strains on PBAT mock hydrolysate. The different evolved strains were cultivated separately in threefold buffered MSM containing a PBAT mock hydrolysate with start concentrations of 80 mM BDO, 40 mM AA, and 40 mM TA. OD_600_ and substrate concentrations were measured at different time points during the cultivation and are shown in black (OD_600_), blue (AA concentration), green (BDO concentration), and purple (TA concentration). The error bars in (**A**) are derived from two replicates, and those in (**B**), (**C**), and (**D**) are from three replicates and indicate the SEM

For evaluation of the potential of this strain for PBAT degradation, all three evolved strains and the unevolved parental strain were cultured on a 2:1:1 monomer mixture of BDO, AA, and TA to mimic a PBAT hydrolysate (Fig. 6). The observed increase in OD_600_ for the unevolved wild type (Fig. 6D) can be attributed to a single outlier within the triplicate, in which the OD_600_ reached approximately 1. As no concurrent consumption of the monomers was detected, this increase is unlikely to reflect biological growth. Instead, it presents an experimental artifact caused by evaporation, likely due to incomplete sealing of the respective culture vessel.

As expected, the TA concentration remained constant for all strains as it is not metabolized. Interestingly, for *G. thermoleovorans* AA, only a minor decrease of AA was observed within 144 h of cultivation. In contrast, BDO was partially consumed (~ 20 mM) (Fig. 6B). This corresponds to the same amount consumed by the strain evolved on BDO, although a considerably lower OD_600_ of 0.8 compared to 2.5 was reached. This indicates that BDO is the favored substrate even when no previous BDO evolution has been conducted.

For the AB strain, a considerably faster growth was observed along with simultaneous consumption of AA and BDO. Additionally, higher OD_600_ values were achieved. Consequently, there were no negative interactions, but rather, a mutual enhancement in the degradation of AA and BDO was observed. While this strain was the only one that fully consumed BDO, AA was not entirely metabolized with 7 mM remaining by the end of cultivation. Nevertheless, *G. thermoleovorans* AB was the only strain that showed considerable utilization of AA. These results underline that the co-metabolizing strain represents a promising alternative to the co-culture approach presented in this study (Fig. 4) and they clearly highlight the potential of this double-evolved strain for the upcycling of PBAT-containing waste, initially aiming at the subsequent purification of the remaining TA, with potential for further upcycling of the metabolized monomers in the future.

These results emphasize that there is still considerable potential for the optimization of the evolved *G. thermoleovorans*, which can be further exploited through evolutionary strain development and targeted genetic engineering.

### Enabling full consumption of adipate

**Fig. 7:**
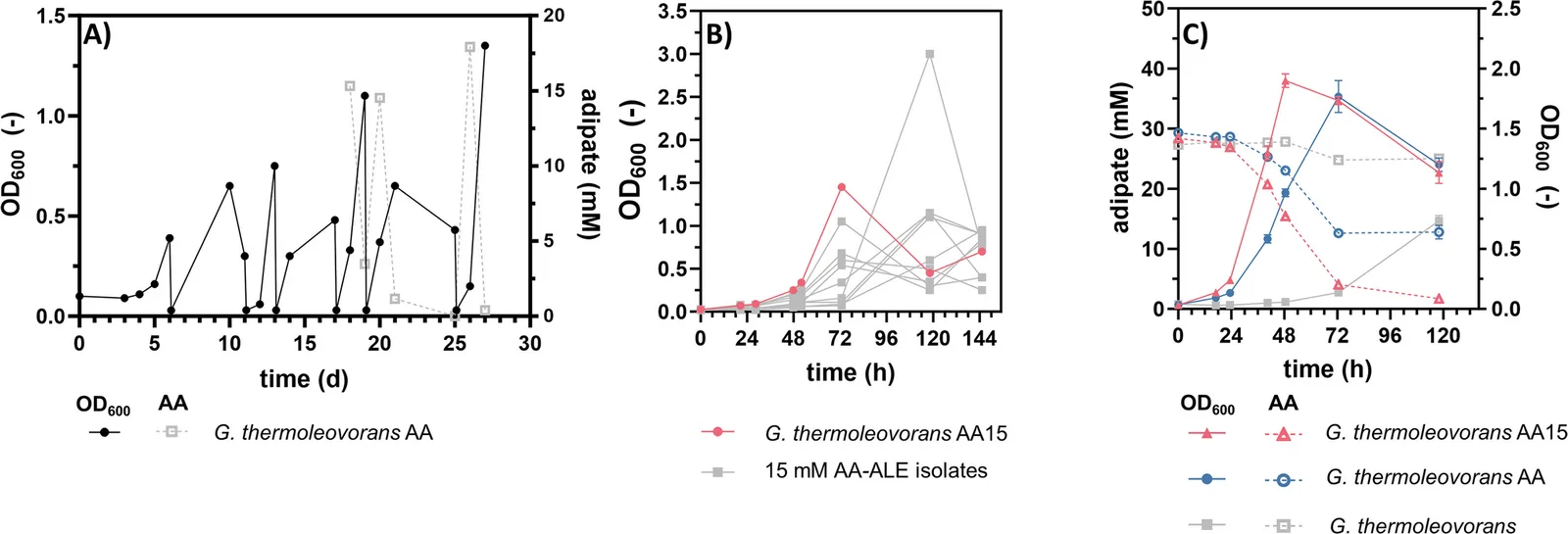
ALE of *G. thermoleovorans* AA on a reduced AA concentration.** A** Sequential batch cultivation of *G. thermoleovorans* AA in 10 mL MSM with 15 mM AA as sole carbon source in Boston bottles. **B** Cultivation of multiple (≥ 6) single clones resulting from the final culture of the ALE on 30 mM AA to select an evolved strain with improved growth. The growth curve of the selected strain (AA15) is shown in red, and those of all the remaining strains are shown in grey. **C** OD_600_ and substrate concentrations measured at different time points during the cultivation of the selected evolved strain (red), the previously evolved strain *G. thermoleovorans* AA (blue), and the unevolved wild type (grey) on 30 mM AA in shake flasks. The substrate concentrations are shown as dashed lines with open symbols. Error bars in (**C**) are derived from three replicates and indicate the SEM

Up to this point, complete metabolization of AA could not be achieved with any of the evolved strains. However, for a potential application of the strains for upcycling of plastic waste, complete substrate degradation is essential to enable simplified purification of remaining monomers or products. Therefore, an additional ALE of the strain already evolved on AA was performed (Fig. 7A) in parallel with the evolution of BDO described above (Fig. 5A). However, the AA concentration applied in this ALE was reduced to the lower limit of the previously observed consumption (15 mM).

From day 18 of the ALE onward, increased OD_600_ values indicated improved growth and potentially enhanced substrate utilization. Therefore, AA concentrations were measured to verify its consumption. This confirmed that AA was completely metabolized by the end of the last two ALE cultures. Several individual clones were isolated and characterized for growth on AA (Fig. 7B). The clone with the highest growth rate, denoted *G. thermoleovorans* AA15, was selected for further experiments.

Direct comparison with the initial strain revealed not only complete metabolization of AA, but also an increased growth rate of approximately 0.1 h^−1^ (Fig. 7C). This indicates that additional mutations occurred during this ALE, which not only confer an advantage at low AA concentrations, but also accelerate growth at higher concentrations. To identify these, the genome was sequenced and the mutations that were additionally identified after this second ALE are listed in Table S12.

One mutation that stands out in particular is the SNV located in the IGR between Gth_000849 and Gth_000850. Interestingly, the neighboring gene cluster (Gth_000850-000860) was upregulated during the growth of *G. thermoleovorans* AA on AA and was therefore identified as relevant for AA metabolization (Fig. 2A, 2C, and 2D). However, in the initial AA degrader, no mutation could be identified in the genomic area of the cluster and no overexpression was detected on glucose. Accordingly, expression of the corresponding genes was likely upregulated in the presence of AA. It is possible that the mutation in strain AA15 changed the gene expression from inducible to constitutive or that it further enhanced expression upon induction. Both effects could increase the growth rate, as the genes might be expressed earlier during cultivation and possibly also more strongly.

This ALE once again highlights the optimization potential that these strains still possess, as well as the value of the applied evolutionary engineering strategy for targeted optimization of non-model organisms.

## Conclusion

In this study, we improved the capacity of *G. thermoleovorans* to metabolize the PBAT-derived monomers AA and BDO through ALE, achieving growth rates of up to 0.10 h^−1^ on AA and 0.13 h^−1^ on BDO. Genome sequencing and transcriptomic analyses of the evolved strains indicated putative metabolic adaptations enhancing uptake and metabolism of both monomers as well as general adaptations to the given culture conditions. The potential of these evolved strains for CBP was shown by cultivation on PBAT as sole carbon source in combination with the HiC cutinase for hydrolysis resulting in growth of the strains on the released AA and BDO. However, incomplete substrate utilization revealed limitations specifically related to the medium composition, underscoring the need for further optimization of both the bioprocess and strain performance. Further ALE-based optimization enabled co-utilization of AA and BDO in one strain, and it also allowed for complete consumption of AA accompanied by improved growth in another. Integration of these improvements into one strain should further enhance its potential for PBAT upcycling.

Altogether, this work illustrates the potential of evolutionary engineering for tailoring non-model thermophiles such as *G. thermoleovorans* to specific biotechnological applications and it provides a high-quality closed genome sequence of this valuable biotechnological chassis. Moreover, it highlights its potential as a promising host for high-temperature CBP of polyester waste. In particular, the engineered strains represent promising candidates for the upcycling of PBAT and related materials, including PBA, PBS, and PBAT blends. The identified pathways can also facilitate the rational design of thermophilic plastic-upcycling hosts by metabolic engineering. Thereby, this work represents an important step toward expanding the process window of plastic-upcycling hosts beyond the widely used *Pseudomonas* species (Ackermann et al. 2021, 2024; de Witt et al. 2023, 2024, 2025; Li et al. 2020; Op de Hipt et al. 2025; Sullivan et al. 2022; Tiso et al. 2021; Welsing et al. 2025; Werner et al. 2025). While these harbor many benefits, such as metabolic versatility, a broad range of genetic engineering tools, and a wide product portfolio, their low-temperature tolerance poses a critical limitation, preventing the implementation of a fully consolidated bioprocess (Op de Hipt et al. 2026). That is why engineering thermophilic CBP hosts is essential. In this context, substantial work is still required to make such a process effective, including the complete metabolism of other plastic monomers, the efficient formation of upcycled products, and possibly also the production and secretion of plastic-hydrolyzing enzymes. This necessitates the development of robust genetic tools for thermophilic hosts, as such production traits are not easily addressed through ALE alone.

## Supplementary Information

Below is the link to the electronic supplementary material.

ESM 1 (PDF 1.61 MB)

## Data Availability

The sequencing data have been deposited in the European Nucleotide Archive under BioProject number PRJEB104702. Additionally, all raw data, computational workflows, and results are accessible as an Annotated Research Context FAIR Digital Object under: 10.60534/y7jxb-5r565. Should any raw data files be needed in another format, they are available from the corresponding author upon reasonable request.
