# Supplementary material for: Evolutionary engineering of Geobacillus thermoleovorans for growth on adipic acid and 1,4-butanediol
Source: Appl Microbiol Biotechnol. 2026 May 2;110:139. doi: 10.1007/s00253-026-13836-8 (PMC13135583; doi:10.1007/s00253-026-13836-8)
Supplement: Supplementary file 1 [file 253_2026_13836_MOESM1_ESM.pdf]

Supplementary data to:

Evolutionary engineering of *Geobacillus thermoleovorans* for growth on adipic acid and 1,4-butanediol

Leonie Op de Hipt[1], Amelie Jäger[1], Tom Luthe[1,5], Volkan Julio Cevik[2], Angela Kranz[2], Benedikt Wynands[1], Julia Frunzke[1,3], Björn Usadel[2,4], Nick Wierckx[1*]

[1]Institute of Bio- and Geosciences IBG-1: Biotechnology, Forschungszentrum Jülich and Biotechnology and Bioeconomy Science Center (BioSC), Jülich, Germany

[2]Institute of Bio- and Geosciences IBG-4: Bioinformatics, Forschungszentrum Jülich, Jülich, Germany

[3]Heinrich Heine University Düsseldorf, Faculty of Mathematics and Natural Sciences, Institute of Microbial Interactions, Düsseldorf, Germany

[4]Heinrich Heine University Düsseldorf, Faculty of Mathematics and Natural Sciences, Institute for Biological Data Science, CEPLAS, Düsseldorf, Germany

[5]Department of Translational and Computational Infection Research (TRACiR), Medical Faculty, Ruhr University Bochum, Bochum, Germany

*Corresponding author:
Nick Wierckx
E-mail: n.wierckx@fz-juelich.de

Table S1: Identified mutations in selected strains derived from the ALE on AA

Affected locus*	Putative function	Mutation (position in genome)**	Putative effect
Gth_000229	EAL-associated domain-containing protein	221312_SNV_C_A	silent mutation L34L
Gth_000444 (*pnp*)	polyribonucleotide nucleotidyltransferase	424522_SNV_C_T	P397S
Intergenic region (IGR) of Gth_000539-Gth_000540	glycerol kinase GlpK; ABC transporter ATP-binding protein	525235_SNV_G_A	alteration of a regulatory region
Gth_001075	AAA family ATPase	1061205_SNV_A_G	silent mutation T153T
IGR of Gth_001152-Gth_001153	peptide MFS transporter; hypothetical protein	1141597_Deletion_A	alteration of a regulatory region
Gth_001339	PBP1A family penicillin-binding protein	multiple breakpoints 1319074-1319173	loss of function
Gth_001651	SAM-dependent methyltransferase	1589967_SNV_G_A	silent mutation N50N
Gth_001747-Gth_001774	Recombinase family protein, hypothetical proteins, ATPase, PTS ascorbate transporter subunit IIC, Fur-regulated basic protein Fbp, primase, helix-turn-helix transcriptional regulator, restriction endonuclease, ImmA/IrrE family metallo-endopeptidase	deletion 1674349-1689977	deletion of 27 coding sequences
IGR of Gth_002031-Gth_002032	ISLre2-like element ISGsp3 family transposase; *rpsD*, 30S ribosomal protein S4	multiple breakpoints 1961378-1961477	alteration of a regulatory region
Gth_002061	DeoR family transcriptional regulator	1993244_SNV_G_A	silent mutation T24T
Gth_002556	hypothetical protein	multiple breakpoints 2471020-2471119	loss of function
Gth_002557	polysaccharide biosynthesis protein	multiple breakpoints 2473169-2473268	loss of function
Gth_003102	IS4 family transposase	multiple breakpoints 3002271-3002370	loss of function
Gth_003191	acyl-CoA dehydrogenase family protein	deletion of 75 bp 3097277-3097351	loss of function
IGR of Gth_003510-Gth_003511	hypothetical protein; ISL3 family transposase	multiple breakpoints 3397140-3397239	alteration of a regulatory region

*Affected loci and **positions refer to gene-IDs in the reference genome available at ENA under the accession number PRJEB104702

Table S2: Identified mutations in selected strains derived from the ALE on BDO

Affected locus*	Putative function	Mutation (position in genome)**	Putative effect
Gth_000444 (*pnp*)	polyribonucleotide nucleotidyltransferase	424517_SNV_G_A	R395H
Gth_000620	ATP-binding protein	629918_SNV_C_T	silent mutation G149G
IGR of Gth_001308-Gth_001309	LysE family transporter//tRNA-Arg	1293435_SNV_A_G	alteration of a regulatory region
IGR of Gth_001308-Gth_001309	LysE family transporter//tRNA-Arg	1293440_SNV_G_A	alteration of a regulatory region
IGR of Gth_001308-Gth_001309	LysE family transporter//tRNA-Arg	1293445_SNV_C_T	alteration of a regulatory region
Gth_001339	PBP1A family penicillin-binding protein	1318921_SNV_A_G	N135S
Gth_001986	metal-dependent hydrolase	1912017_SNV_T_C	D56G
Gth_002032 (*rpsD*)	30S ribosomal protein S4	1961907_SNV_G_A	R100H
Gth_002057	NERD domain-containing protein	1989423_SNV_A_G	silent mutation R78R

Gth_003123	RNA-guided endonuclease InsQ/TnpB family protein	3025148_SNV_G_A	silent mutation T119T
Gth_003123	RNA-guided endonuclease InsQ/TnpB family protein	3025170_SNV_T_C	S127P
IGR of Gth_003123-Gth_003124	RNA-guided endonuclease InsQ/TnpB family protein//aminotransferase class I/II-fold pyridoxal phosphate-dependent enzyme	3025937_Deletion_T_-	alteration of a regulatory region
IGR of Gth_003123-Gth_003124	RNA-guided endonuclease InsQ/TnpB family protein//aminotransferase class I/II-fold pyridoxal phosphate-dependent enzyme	3025939_SNV_C_G	alteration of a regulatory region
IGR of Gth_003123-Gth_003124	RNA-guided endonuclease InsQ/TnpB family protein//aminotransferase class I/II-fold pyridoxal phosphate-dependent enzyme	3025945_SNV_G_A	alteration of a regulatory region
Gth_003140 (*hutH*)	histidine ammonia-lyase	3045012_SNV_T_A	silent mutation A445A

*Affected loci and **positions refer to gene-IDs in the reference genome available at ENA under the accession number PRJEB104702

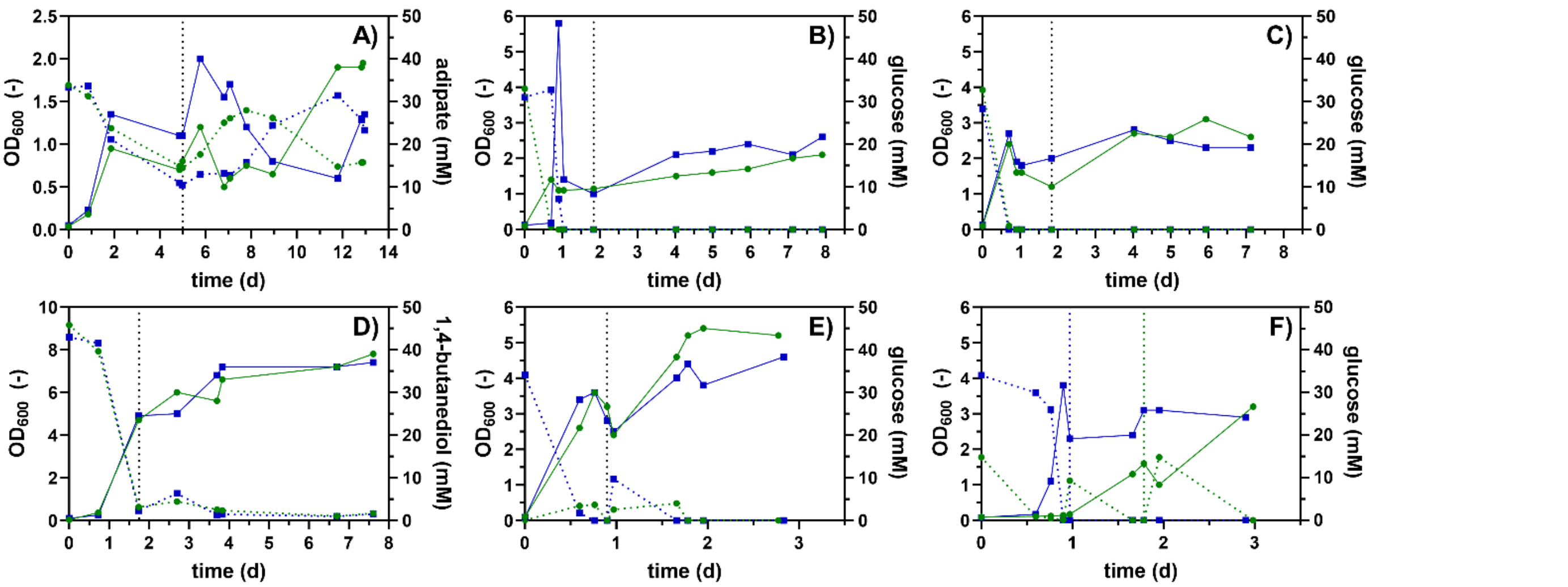

Figure S1: Chemostat cultures of the evolved *G. thermoleovorans* strains for transcriptomic analysis. The strain evolved on AA (*G. thermoleovorans* AA) was cultivated at a dilution rate of 0.04 h^{-1} on 30 mM AA (**A**) and 30 mM glucose (**B**). The strain evolved on BDO (*G. thermoleovorans* BDO) was cultivated at a dilution rate of 0.15 h^{-1} on 45 mM BDO (**D**) and 30 mM glucose (**E**). The unevolved wild type (*G. thermoleovorans*) was cultivated on 30 mM glucose at a dilution rate of 0.04 h^{-1} (**C**) and 0.15 h^{-1} (**F**). OD_{600} values (solid lines) and substrate concentrations (dashed lines) are plotted against time. The time of the switch from batch to continuous process mode is indicated by a dashed vertical line. All cultivations were carried out in duplicates with one duplicate shown in green and one in blue

Table S3: Upregulated genes during growth of *G. thermoleovorans* AA on AA compared to the wild type on glucose. A color code represents the values of $\log_2$ fold change and $-\log_{10}$(FDR p-value) with stepwise increasing darkness of the color for the following levels: 0-3.99; 4-7.99; 8-11.99; 12-16

Locus*	$\log_2$ fold change	$-\log_{10}$(FDR p-value)	Function
Gth_000178	3.10	8.63	FixH family protein
Gth_000211	1.17	2.28	NAD(P)-dependent oxidoreductase
Gth_000214	1.46	3.08	long-chain fatty acid-CoA ligase
Gth_000584	1.67	3.98	glucose 1-dehydrogenase
Gth_000586	2.99	9.74	acyl-CoA dehydrogenase family protein
Gth_000692	1.92	4.32	IclR family transcriptional regulator
Gth_000693 (*pxpB*)	1.90	5.97	5-oxoprolinase subunit PxpB
Gth_000694	1.76	5.23	biotin-dependent carboxyltransferase family protein
Gth_000695	1.71	5.78	5-oxoprolinase subunit PxpA
Gth_000815	1.51	2.34	GntR family transcriptional regulator
Gth_000816	1.72	5.40	ABC transporter ATP-binding protein
Gth_000817	1.43	2.70	ABC transporter permease
Gth_000850	5.10	13.93	acyl-CoA dehydrogenase family protein
Gth_000851	5.76	16	beta-ketoacyl-ACP reductase
Gth_000852	5.90	16	MaoC family dehydratase N-terminal domain-containing protein
Gth_000853	6.02	16	MaoC family dehydratase
Gth_000854	5.87	16	acetyl-CoA C-acyltransferase
Gth_000855	5.94	16	TetR/AcrR family transcriptional regulator
Gth_000856	5.02	16	branched-chain amino acid ABC transporter permease
Gth_000857	4.99	16	branched-chain amino acid ABC transporter permease
Gth_000858	6.30	16	ABC transporter ATP-binding protein
Gth_000859	6.22	16	ABC transporter ATP-binding protein
Gth_000860	6.51	16	ABC transporter substrate-binding protein
Gth_000932	2.437	4.86	DUF6044 family protein
Gth_000933	3.11	10.2	transporter, sodium/bile acid symporter family protein
Gth_000934 (*pcp*)	3.35	12.64	pyroglutamyl-peptidase I
Gth_000935	3.46	16	DUF979 domain-containing protein
Gth_000936	3.41	11.27	DUF969 domain-containing protein
Gth_001220	1.60	2.05	ABC transporter permease subunit
Gth_001221	1.74	2.59	nitrous oxide reductase accessory protein NosL
Gth_001222	2.05	3.94	nitrous oxide reductase family maturation protein NosD
Gth_001296	3.03	4.79	VOC family protein
Gth_001297	2.41	5.79	YhfC family intramembrane metalloprotease
Gth_001299	1.77	2.28	RNA-guided endonuclease InsQ/TnpB family protein
Gth_001300	1.97	3.83	carboxyl transferase domain-containing protein
Gth_001301	2.27	5.23	AMP-binding protein
Gth_002079 (*pckA*)	5.75	16	phosphoenolpyruvate carboxykinase
Gth_002110	1.36	2.43	glycogen/starch/alpha-glucan phosphorylase
Gth_002111 (*glgA*)	2.11	3.92	glycogen synthase GlgA
Gth_002112	1.97	2.59	sugar phosphate nucleotidyltransferase
Gth_002113	2.01	4.45	glucose-1-phosphate adenylyltransferase
Gth_002242	1.81	2.15	biotin transporter BioY
Gth_002247	1.33	2.96	ABC transporter ATP-binding protein
Gth_002248	1.34	2.59	branched-chain amino acid ABC transporter permease
Gth_002249	1.36	2.98	branched-chain amino acid ABC transporter permease
Gth_002250	1.23	3.37	ABC transporter substrate-binding protein
Gth_002251	1.36	2.97	GntR family transcriptional regulator
Gth_002252 (*hpaE*)	2.027	4.25	5-carboxymethyl-2-hydroxymuconate semialdehyde dehydrogenase
Gth_002253	2.28	6.96	5-carboxymethyl-2-hydroxymuconate Delta-isomerase
Gth_002254	2.26	7.05	fumarylacetoacetate hydrolase family protein
Gth_002255	2.12	4.59	fumarylacetoacetate hydrolase family protein
Gth_002256 (*hpaI*)	2.31	7.47	2,4-dihydroxyhept-2-ene-1,7-dioic acid aldolase
Gth_002257 (*hpaD*)	2.43	5.95	3,4-dihydroxyphenylacetate 2,3-dioxygenase
Gth_002258 (*hpaB*)	2.65	11.94	4-hydroxyphenylacetate 3-monooxygenase. oxygenase component
Gth_002260	3.49	7.32	manganese catalase family protein
Gth_002261	4.24	6.54	YuzF family protein
Gth_002262	4.40	8.02	PepSY-associated TM helix domain-containing protein
Gth_002264	1.37	2.13	DUF6230 family protein
Gth_002265	1.52	2.27	DUF6114 domain-containing protein
Gth_002355	2.20	4.76	glycosyltransferase family 4 protein
Gth_002356	2.13	6.30	6-hydroxymethylpterin diphosphokinase MptE-like protein
Gth_002357	2.07	6.26	UDP-N-acetylglucosamine 4,6-dehydratase family protein
Gth_002358	2.19	4.76	hypothetical protein
Gth_002359	2.86	7.18	flagellin
Gth_002692 (*qoxD*)	2.70	6.60	cytochrome aa3 quinol oxidase subunit IV
Gth_002693 (*qoxC*)	3.08	10.58	cytochrome aa3 quinol oxidase subunit III
Gth_002694 (*qoxB*)	3.88	16	cytochrome aa3 quinol oxidase subunit I
Gth_002695 (*qoxA*)	4.46	16	cytochrome aa3 quinol oxidase subunit II
Gth_002945	7.32	16	sigma 54-interacting transcriptional regulator
Gth_002946	7.11	16	acyl-CoA dehydrogenase family protein
Gth_002947	2.36	4.16	hypothetical protein
Gth_002948	2.30	4.10	aldehyde dehydrogenase family protein
Gth_002949	2.28	4.33	iron-containing alcohol dehydrogenase
Gth_002950	1.80	2.44	DoxX family protein
Gth_003095	1.66	2.57	hypothetical protein
Gth_003096	1.56	2.24	SPFH domain-containing protein
Gth_003112	2.76	11.34	sugar phosphate nucleotidyltransferase
Gth_003113	2.80	9.56	glycosyltransferase family 4 protein
Gth_003114	2.75	11.33	glycosyl transferase

Gth_003115	2.64	9.64	phosphomannomutase/phosphoglucomutase
Gth_003120	1.35	2.10	RDD family protein
Gth_003124	5.55	16	aminotransferase class I/II-fold pyridoxal phosphate-dependent enzyme
Gth_003125	6.27	16	thiamine pyrophosphate-binding protein
Gth_003126	6.76	16	aldehyde dehydrogenase family protein
Gth_003127	6.82	16	saccharopine dehydrogenase C-terminal domain-containing protein
Gth_003179	2.64	7.93	flagellar basal body rod protein
Gth_003180	2.57	7.96	PspA/IM30 family protein
Gth_003184	1.53	2.71	DUF1640 domain-containing protein
Gth_003186	0.99	2.34	Gth_003186
Gth_003190	3.47	16	DUF3870 domain-containing protein
Gth_003191	6.71	16	acyl-CoA dehydrogenase family protein
Gth_003192	7.50	16	CoA transferase
Gth_003196	2.08	3.46	GNAT family protein
Gth_003197	2.02	4.86	TIGR01777 family oxidoreductase
Gth_003213	1.80	3.98	DUF402 domain-containing protein
Gth_003214	1.64	2.87	ABC transporter ATP-binding protein
Gth_003215	1.74	4.19	ABC transporter ATP-binding protein
Gth_003216	2.12	3.89	ABC transporter substrate-binding protein
Gth_003217	1.71	4.78	ABC transporter permease
Gth_003218 (*nikC*)	1.41	2.40	nickel transporter permease
Gth_003219	1.54	3.88	aromatic acid exporter family protein
Gth_003220	2.71	7.62	glutamate synthase
Gth_003221	1.15	2.18	glutamate-1-semialdehyde 2,1-aminomutase
Gth_003232	1.19	2.02	YgzB family protein
Gth_003233	1.85	5.55	nucleotidyltransferase-like protein
Gth_003262	2.01	3.65	PrkA family serine protein kinase
Gth_003263 (*yhbH*)	2.16	7.17	sporulation protein YhbH
Gth_003264	1.51	2.23	hypothetical protein
Gth_003266	1.22	2.46	hypothetical protein
Gth_003270	3.83	16	dicarboxylate/amino acid:cation symporter
Gth_003275	1.38	3.35	YhcU family protein
Gth_003280	2.10	5.93	phospho-sugar mutase
Gth_003281	1.84	2.13	YhdB family protein
Gth_003284	1.27	1.66	sensor histidine kinase
Gth_003285	1.87	3.94	response regulator transcription factor
Gth_003286	3.20	10.64	VTT domain-containing protein
Gth_003287	3.50	12.80	undecaprenyl-diphosphatase
Gth_003288	3.85	16	SpoVR family protein
Gth_003289	1.88	3.94	RNA-binding domain-containing protein
Gth_003292	4.08	3.42	helix-turn-helix domain-containing protein
Gth_003297	2.60	6.42	AAA family ATPase
Gth_003311	1.79	5.55	IS66-like element ISBst12 family transposase
Gth_003312	2.26	5.57	DNRLRE domain-containing protein
Gth_003423	2.16	3.64	competence protein ComK
Gth_003424	2.31	6.14	hypothetical protein
Gth_003496	3.21	10.40	cytochrome ubiquinol oxidase subunit I
Gth_003529	2.34	5.35	PCYCGC domain-containing protein
Gth_003534	2.63	5.97	FTR1 family protein
Gth_003536	1.88	3.25	TOPRIM nucleotidyl transferase/hydrolase domain-containing protein

*Loci refer to GenIDs in the reference genome available at NCBI under the accession number PRJEB104702

Table S4: Downregulated genes during growth of *G. thermoleovorans* AA on AA compared to the wild type on glucose. A color code represents the values of $\log_2$ fold change and $-\log_{10}$(FDR p-value) with stepwise increasing darkness of the color for the following levels: 0- |3.99 |; |4|-|7.99|; |8|-|11.99|; |12|-|16|

Locus*	Log2 fold change	-log10(FDR p-value)	Function
Gth_000199	-4.55	5.23	transcription antiterminator
Gth_000200 (*ptsG*)	-8.61	16	glucose-specific PTS transporter subunit IIBC
Gth_000201	-5.13	16	phosphocarrier protein HPr
Gth_000202 (*ptsP*)	-4.20	10.84	phosphoenolpyruvate-protein phosphotransferase
Gth_000205	-5.35	8.23	YbxH family protein
Gth_000331 (*pyrF*)	-1.34	2.36	orotidine-5'-phosphate decarboxylase
Gth_000332 (*pyre*)	-1.22	3.10	orotate phosphoribosyltransferase
Gth_001155	-1.65	2.2	alpha-ketoacid dehydrogenase subunit beta
Gth_001156 (*pdhA*)	-2.57	4.19	pyruvate dehydrogenase (acetyl-transferring) E1component subunit alpha
Gth_001157	-2.36	3.39	ABC transporter ATP-binding protein
Gth_001158	-2.57	3.99	ABC transporter permease
Gth_001159	-2.83	6.40	ABC transporter substrate-binding protein
Gth_001160	-2.3	4.00	Leu/Phe/Val dehydrogenase
Gth_001177	-2.17	2.1	glutamate-5-semialdehyde dehydrogenase
Gth_001178	-2.68	3.74	beta-eliminating lyase-related protein
Gth_001179	-3.56	6.98	GtrA family protein
Gth_001180	-3.97	7.48	glycosyltransferase family 2 protein
Gth_001181	-4.32	6.97	DUF3488 domain-containing protein
Gth_001188	-1.51	2.66	MFS transporter
Gth_001189	-1.92	2.78	TRAP transporter large permease subunit
Gth_001190	-2.37	3.98	TRAP transporter small permease
Gth_001191	-2.26	3.94	TRAP transporter substrate-binding protein
Gth_001192	-3.21	5.00	sugar phosphate isomerase/epimerase family protein

Gth_001193	-3.19	4.94	thiamine pyrophosphate-binding protein
Gth_001194	-315	4.11	alcohol dehydrogenase catalytic domain-containing protein
Gth_001195	-3.39	6.73	aldehyde dehydrogenase family protein
Gth_001196	-3.27	3.66	citryl-CoA lyase
Gth_001277	-1.62	2.13	Gfo/Idh/MocA family oxidoreductase
Gth_001278	-1.70	2.81	trehalose utilization protein ThuA
Gth_001279	-1.59	2.24	Gfo/Idh/MocA family oxidoreductase
Gth_001747	-12.39	4.42	recombinase family protein
Gth_001748	-8.90	2.20	hypothetical protein
Gth_001749	-12.31	4.38	hypothetical protein
Gth_001750	-13.40	5.28	hypothetical protein
Gth_001751	-13.51	5.38	ATPase
Gth_001752	-13.12	5.05	hypothetical protein
Gth_001753	-13.09	5.03	hypothetical protein
Gth_001755	-12.13	4.22	hypothetical protein
Gth_001756	-13.88	5.71	hypothetical protein
Gth_001757	-15.66	7.39	hypothetical protein
Gth_001758	-16.01	7.69	hypothetical protein
Gth_001759	-12.48	4.55	hypothetical protein
Gth_001760	-13.68	5.48	hypothetical protein
Gth_001761	-11.58	3.86	PTS ascorbate transporter subunit IIC
Gth_001764	-12.28	4.33	hypothetical protein
Gth_001766	-10.10	2.78	hypothetical protein
Gth_001767	-15.15	6.84	phage/plasmid primase, P4 family
Gth_001768	-14.40	6.15	hypothetical protein
Gth_001770	-11.45	3.73	hypothetical protein
Gth_001771	-13.97	5.74	helix-turn-helix transcriptional regulator
Gth_001772	-11.87	4.07	helix-turn-helix transcriptional regulator
Gth_001773	-13.49	5.35	restriction endonuclease
Gth_001774	-12.82	4.81	ImmA/IrrE family metallo-endopeptidase
Gth_001988 (*ald*)	-3.3	16	alanine dehydrogenase
Gth_002084	-1.63	2.44	ABC transporter substrate-binding protein
Gth_002086	-2.02	3.17	ABC transporter permease
Gth_002087 (*ytkD*)	-1.84	2.83	RNA deprotection pyrophosphohydrolase
Gth_002088	-2.28	3.42	hydrolase
Gth_002089	-2.00	2.64	holin family protein
Gth_002444	-5.52	16	carbohydrate ABC transporter permease
Gth_002445	-5.15	16	carbohydrate ABC transporter permease
Gth_002446	-5.33	16	ABC transporter substrate-binding protein
Gth_002554	-2.31	16	hypothetical protein
Gth_002556	-1.76	16	hypothetical protein
Gth_002557	-1.60	16	polysaccharide biosynthesis protein
Gth_002673	-1.95	2.76	FMN-dependent NADH-azoreductase
Gth_002678	-1.65	2.64	glycoside hydrolase family 32 protein
Gth_002679	-1.54	2.50	sucrose-specific PTS transporter subunit IIBC
Gth_002923	-2.01	2.79	8-oxo-dGTP diphosphatase
Gth_002924	-1.90	5.01	IS110 family transposase
Gth_003082	-3.53	3.16	PspA/IM30 family protein
Gth_003083	-3.69	3.40	hypothetical protein
Gth_003086	-2.61	3.08	ATP-binding protein
Gth_003087	-2.66	4.60	response regulator transcription factor
Gth_003088	-3.68	3.98	trypsin-like peptidase domain-containing protein
Gth_003470	-1.88	2.95	ABC transporter ATP-binding protein
Gth_003474	-1.81	2.84	thiazole biosynthesis adenylyltransferase ThiF
Gth_003475	-1.90	4.90	thiazole synthase
Gth_003476 (*thiS*)	-2.36	5.23	sulfur carrier protein ThiS
Gth_003477 (*thiO*)	-2.10	4.04	glycine oxidase ThiO
Gth_003478 (*tenI*)	-2.18	4.33	thiazole tautomerase TenI
Gth_003479	-1.90	4.93	energy-coupling factor transporter transmembrane protein EcfT
Gth_003480	-1.92	2.49	ABC transporter ATP-binding protein
Gth_003481	-1.86	5.00	ECF transporter S component
Gth_003482 (*tenA*)	-1.68	4.09	thiaminase II

*Loci refer to GenIDs in the reference genome available at NCBI under the accession number PRJEB104702

Table S5: Upregulated genes during growth of *G. thermoleovorans* AA on glucose compared to the wild type on glucose. A color code represents the values of $\log_2$ fold change and $-\log_{10}$(FDR p-value) with stepwise increasing darkness of the color for the following levels: 0-3.99; 4-7.99; 8-11.99; 12 -16

Locus*	$\mathbf{Log_2}$ fold change	$\mathbf{-log_{10}}$(FDR p-value)	Function
Gth_000106	2.92	2.71	sulfite exporter TauE/SafE family protein
Gth_000699	1.83	2.58	long-chain fatty acid-CoA ligase
Gth_000700	1.93	3.80	Phosphotriesterase
Gth_000701	2.65	2.02	hypothetical protein
Gth_000860	1.83	3.54	ABC transporter substrate-binding protein
Gth_001191	2.22	2.75	TRAP transporter substrate-binding protein
Gth_001193	2.50	2.67	thiamine pyrophosphate-binding protein
Gth_001194	2.68	2.94	alcohol dehydrogenase catalytic domain-containing protein
Gth_001195	2.65	3.38	aldehyde dehydrogenase family protein
Gth_001196	2.77	2.74	citryl-CoA lyase
Gth_002000	1.96	2.07	DUF6230 family protein
Gth_002004	1.72	2.94	Zn-dependent alcohol dehydrogenase
Gth_002006	1.67	2.06	AarF/UbiB family protein
Gth_002007	1.82	2.34	acetyl-CoA C-acyltransferase

Gth_002008	1.72	3.35	SDR family NAD(P)-dependent oxidoreductase
Gth_002009	1.75	4.14	enoyl-CoA hydratase/isomerase family protein
Gth_002010	1.79	3.54	acyl-CoA dehydrogenase family protein
Gth_002011	1.68	3.26	acyl-CoA dehydrogenase family protein
Gth_002012	1.58	3.72	long-chain fatty acid-CoA ligase
Gth_002091 (*ytzI*)	2.32	2.69	YtzI protein
Gth_002706	4.03	2.38	ISLre2-like element ISGsp3 family transposase
Gth_002945	5.84	16	sigma 54-interacting transcriptional regulator
Gth_002946	5.24	16	acyl-CoA dehydrogenase family protein
Gth_003124	3.61	10.33	aminotransferase class I/II-fold pyridoxal phosphate-dependent enzyme
Gth_003125	4.41	12.96	thiamine pyrophosphate-binding protein
Gth_003126	4.76	16	aldehyde dehydrogenase family protein
Gth_003127	4.65	16	saccharopine dehydrogenase C-terminal domain-containing protein
Gth_003190	2.75	8.85	DUF3870 domain-containing protein
Gth_003191	5.44	16	acyl-CoA dehydrogenase family protein
Gth_003192	6.11	16	CoA transferase
Gth_003270	1.42	2.26	dicarboxylate/amino acid:cation symporter
Gth_003292	3.92	2.28	helix-turn-helix domain-containing protein
Gth_003375	1.58	2.62	IS701 family transposase
Gth_003448	1.40	3.12	YhaI family protein
Gth_003371 (*adhP*)	3.84	2.71	alcohol dehydrogenase AdhP
Gth_003372 (*adh*)	3.93	2.80	aldehyde dehydrogenase

*Loci refer to GenIDs in the reference genome available at NCBI under the accession number PRJEB104702

Table S6: Downregulated genes during growth of *G. thermoleovorans* AA on glucose compared to the wild type on glucose. A color code represents the values of $\log_2$ fold change and $-\log_{10}$(FDR p-value) with stepwise increasing darkness of the color for the following levels: 0-|3.99|; |4|-|7.99|; |8|-|11.99|; |12|-|16|

**Locus***	**$\log_2$ fold change**	**$-\log_{10}$(FDR p-value)**	**Function**
Gth_000153	-1.94	2.14	ABC transporter permease
Gth_000154	-1.94	2.34	sugar ABC transporter ATP-binding protein
Gth_000650	-2.75	5.20	ABC transporter substrate-binding protein
Gth_000667	-3.06	2.94	phosphopantetheine-binding protein
Gth_000669	-2.58	2.02	HAMP domain-containing protein
Gth_000670	-2.52	2.56	STAS domain-containing protein
Gth_000671	-2.41	2.34	PP2C family protein-serine/threonine phosphatase
Gth_000673	-2.70	2.63	STAS domain-containing protein
Gth_000674	-2.39	2.83	LytR/AlgR family response regulator transcription factor
Gth_000707	-2.22	3.54	acyltransferase family protein
Gth_001179	-3.00	3.60	GtrA family protein
Gth_001180	-3.30	4.10	glycosyltransferase family 2 protein
Gth_001181	-3.35	3.40	DUF3488 domain-containing protein
Gth_001747	-11.90	3.08	recombinase family protein
Gth_001749	-11.82	3.05	hypothetical protein
Gth_001750	-12.91	3.69	hypothetical protein
Gth_001751	-13.02	3.74	ATPase
Gth_001752	-12.63	3.54	hypothetical protein
Gth_001753	-12.60	3.52	hypothetical protein
Gth_001755	-11.64	2.94	hypothetical protein
Gth_001756	-13.39	4.02	hypothetical protein
Gth_001757	-15.17	5.37	hypothetical protein
Gth_001758	-15.52	5.62	hypothetical protein
Gth_001759	-11.99	3.14	hypothetical protein
Gth_001760	-13.19	3.80	hypothetical protein
Gth_001761	-11.10	2.58	PTS ascorbate transporter subunit IIC
Gth_001764	-11.79	3.00	hypothetical protein
Gth_001767	-14.66	4.93	phage/plasmid primase, P4 family
Gth_001768	-13.92	4.25	hypothetical protein
Gth_001770	-10.96	2.47	hypothetical protein
Gth_001771	-13.49	4.03	helix-turn-helix transcriptional regulator
Gth_001772	-9.25	8.42	helix-turn-helix transcriptional regulator
Gth_001773	-13.00	3.74	restriction endonuclease
Gth_001774	-12.33	3.34	ImmA/IrrE family metallo-endopeptidase
Gth_001921	-2.43	4.08	hypothetical protein
Gth_002119	-1.96	2.04	Ig-like domain-containing protein
Gth_002509	-2.11	2.04	carbohydrate ABC transporter permease
Gth_002540	-4.27	12.20	IS4 family transposase
Gth_002553	-3.09	3.68	hypothetical protein
Gth_002554	-4.11	4.68	hypothetical protein
Gth_002556	-2.94	3.93	hypothetpolysaccharide biosynthesis proteinical protein
Gth_002557	-2.47	4.08	polysaccharide biosynthesis protein
Gth_002941	-1.96	2.04	FecCD family ABC transporter permease
Gth_003086	-2.43	2.07	ATP-binding protein
Gth_003087	-2.32	2.94	response regulator transcription factor
Gth_003088	-3.22	2.36	trypsin-like peptidase domain-containing protein
Gth_003550	-2.01	2.08	glycoside hydrolase family 130 protein
Gth_003594	-2.66	2.71	hypothetical protein
Gth_003595	-2.64	2.21	hypothetical protein

*Loci refer to GenIDs in the reference genome available at NCBI under the accession number PRJEB104702

Table S7: Upregulated genes during growth of *G. thermoleovorans* BDO on BDO compared to the wild type on glucose. A color code represents the values of log_2 fold change and $-log_{10}$(FDR p-value) with stepwise increasing darkness of the color for the following levels: 0-3.99; 4-7.99; 8-11.99; 12 -16.

Locus*	Log₂ fold change	-log₁₀(FDR p-value)	Function
Gth_000017	2.24	2.05	hypothetical protein
Gth_000018	4.73	9.88	hypothetical protein
Gth_000052	2.71	2.32	CotS family spore coat protein
Gth_000053	2.45	2.16	glycosyltransferase family 4 protein
Gth_000056	2.32	2.44	Glycosyltransferase
Gth_000057	3.34	5.73	Phosphotransferase
Gth_000058	4.32	8.91	hypothetical protein
Gth_000062	4.38	8.67	hypothetical protein
Gth_000064	3.27	4.90	hypothetical protein
Gth_000065	2.46	2.82	Holing
Gth_000129	3.07	4.47	DUF5667 domain-containing protein
Gth_000192	2.62	3.10	hypothetical protein
Gth_000222	1.77	2.07	glycoside hydrolase domain-containing protein
Gth_000248	4.24	2.32	GapA-binding peptide SR1P
Gth_000310 (*spoIIGA*)	2.07	2.51	sigma-E processing peptidase SpoIIGA
Gth_000313	2.76	3.61	YlmC/YmxH family sporulation protein
Gth_000447	2.86	2.18	YlmC/YmxH family sporulation protein
Gth_000449	4.17	7.81	dipicolinate synthase subunit B
Gth_000448 (*dpaA*)	3.94	8.12	dipicolinic acid synthetase subunit A
Gth_000482	3.46	5.73	outer spore coat protein CotE
Gth_000586	4.46	8.09	acyl-CoA dehydrogenase family protein
Gth_000603	1.75	2.03	hypothetical protein
Gth_000676	2.28	2.32	FeoB small GTPase domain-containing protein
Gth_000677	3.35	5.32	FeoA family protein
Gth_000771	2.39	2.18	hypothetical protein
Gth_000829	2.67	3.57	hypothetical protein
Gth_000962	2.46	2.45	NUDIX domain-containing protein
Gth_000964	4.28	8.12	DUF4183 domain-containing protein
Gth_001009	2.29	2.25	hypothetical protein
Gth_001044	7.15	16	iron-containing alcohol dehydrogenase
Gth_001045	7.09	16	N-acyl homoserine lactonase family protein
Gth_001049 (*xylB*)	2.75	2.97	Xylulokinase
Gth_001050 (*xylA*)	3.48	6.34	xylose isomerase
Gth_001051	3.06	5.08	aldose epimerase family protein
Gth_001052	3.07	4.13	Gfo/Idh/MocA family oxidoreductase
Gth_001053	3.40	6.07	sugar ABC transporter permease
Gth_001054	3.90	6.18	xylose ABC transporter ATP-binding protein
Gth_001055 (*xylF*)	3.75	4.33	D-xylose ABC transporter substrate-binding protein
Gth_001064	2.38	2.21	ABC transporter permease
Gth_001070	2.38	2.96	LacI family DNA-binding transcriptional regulator
Gth_001071	3.37	6.66	alpha-mannosidase
Gth_001072	2.56	2.14	carbohydrate ABC transporter permease
Gth_001073	2.66	4.02	DUF6431 domain-containing protein
Gth_001074	2.49	3.02	IS481 family transposase
Gth_001075	12.38	2.61	AAA family ATPase
Gth_001076	3.28	4.29	carbohydrate ABC transporter permease
Gth_001077	3.50	6.79	carbohydrate ABC transporter permease
Gth_001078	3.79	7.13	extracellular solute-binding protein
Gth_001079	3.40	5.35	substrate-binding domain-containing protein
Gth_001080	2.78	3.26	enoyl-CoA hydratase
Gth_001081	2.18	3.04	ArnT family glycosyltransferase
Gth_001082	2.58	2.82	aldehyde dehydrogenase family protein
Gth_001083	2.23	2.32	cyclase family protein
Gth_001084	3.11	5.09	DapH/DapD/GlmU-related protein
Gth_001086	3.07	7.31	D-serine ammonia-lyase
Gth_001221	2.11	2.12	nitrous oxide reductase accessory protein NosL
Gth_001222	2.44	2.19	nitrous oxide reductase family maturation protein NosD
Gth_001299	4.16	6.90	RNA-guided endonuclease InsQ/TnpB family protein
Gth_001329	3.35	4.15	Hsp20 family protein
Gth_001333	2.32	2.32	YppG family protein
Gth_001409	3.30	4.52	spore coat associated protein CotJA
Gth_001410	3.12	4.96	spore coat protein CotJB
Gth_001411	2.25	2.44	manganese catalase family protein
Gth_001457	2.70	3.39	superoxide dismutase
Gth_001684	3.90	5.24	DUF2624 domain-containing protein
Gth_001687 (*vrrA*)	3.57	5.44	VrrA/YqfQ family protein
Gth_001859	1.66	2.08	stage II sporulation protein D
Gth_001879 (*ysxE*)	2.07	2.18	spore coat protein YsxE
Gth_001880 (*spoVID*)	2.13	2.20	stage VI sporulation protein D
Gth_001908	4.27	8.26	LuxR C-terminal-related transcriptional regulator
Gth_001963	5.18	13.11	glyceraldehyde-3-phosphate dehydrogenase
Gth_002079	5.49	16	phosphoenolpyruvate carboxykinase (ATP)
Gth_002084	2.66	4.47	ABC transporter substrate-binding protein
Gth_002085	2.42	3.32	ABC transporter ATP-binding protein
Gth_002260	3.30	4.06	manganese catalase family protein
Gth_002261	3.66	4.66	YuzF family protein
Gth_002573 (*spoIIID*)	3.17	3.80	sporulation transcriptional regulator SpoIIID
Gth_002661	2.23	2.31	cell wall hydrolase
Gth_002798 (*yabP*)	2.08	2.61	sporulation protein YabP
Gth_002960	4.04	7.63	S8 family serine peptidase
Gth_003112	2.28	2.51	sugar phosphate nucleotidyltransferase

Gth_003131	2.38	2.21	xanthine dehydrogenase family protein molybdopterin-binding subunit
Gth_003133	2.14	2.18	XdhC family protein
Gth_003134	2.43	2.32	hypothetical protein
Gth_003136	2.99	3.39	hypothetical protein
Gth_003150 (*mreBH*)	3.06	3.39	rod-share determining protein MreBH
Gth_003400 (*asnB*)	2.44	2.43	asparagine synthase (glutamine-hydrolyzing)
Gth_003410	5.08	6.09	spore germination protein
Gth_003411	4.74	7.45	spore germination protein GerPB
Gth_003460	2.68	3.83	Cof-type HAD-IIB family hydrolase
Gth_003463	2.69	2.47	YheC/YheD family protein
Gth_003472	3.22	3.78	small, acid-soluble spore protein, alpha/beta type
Gth_003497	2.31	2.32	SpoVR family protein
Gth_003560	2.95	2.68	L,D-transpeptidase family protein
Gth_003601	2.45	2.72	IS1634 family transposase

*Loci refer to GenIDs in the reference genome available at NCBI under the accession number PRJEB104702

Table S8: Downregulated genes during growth of *G. thermoleovorans* BDO on BDO compared to the wild type on glucose. A color code represents the values of $\log_2$ fold change and $-\log_{10}$(FDR p-value) with stepwise increasing darkness of the color for the following levels: 0-ǀ3.99ǀ; ǀ4ǀ-ǀ7.99ǀ; ǀ8ǀ-ǀ11.99ǀ; ǀ12ǀ-ǀ16ǀ

Locus*	$\log_2$ fold change	$-\log_{10}$(FDR p-value)	Function
Gth_000137	-1.89	2.20	DUF485 domain-containing protein
Gth_000138	-3.58	5.38	amino acid permease
Gth_000169	-2.75	3.56	amino acid permease
Gth_000177	-3.71	6.88	AAA family ATPase
Gth_000193	-2.30	2.61	glucose 1-dehydrogenase
Gth_000200 (*ptsg*)	-7.71	16	glucose-specific PTS transporter subunit IIBC
Gth_000201	-4.57	8.67	phosphocarrier protein HPr
Gth_000202 (*ptsP*)	-4.00	7.45	phosphoenolpyruvate-protein phosphotransferase
Gth_000325	-3.24	2.52	aspartate carbamoyltransferase catalytic subunit
Gth_000326	-2.85	2.67	dihydroorotase
Gth_000327	-2.69	2.05	carbamoyl phosphate synthase small subunit
Gth_000381	-2.02	3.60	flagellar hook-length control protein FliK
Gth_000646	-7.20	8.66	ammonium transporter
Gth_000647	-7.60	9.00	DUF294 nucleotidyltransferase-like domain-containing protein
Gth_000648	-6.98	7.74	3'-5' exonuclease
Gth_000666	-2.56	2.20	alpha/beta hydrolase
Gth_000668	-2.86	2.73	class I adenylate-forming enzyme family protein
Gth_000669	-2.69	3.12	HAMP domain-containing protein
Gth_000670	-2.61	2.68	STAS domain-containing protein
Gth_000671	-2.80	3.64	PP2C family protein-serine/threonine phosphatase
Gth_000672	-2.77	3.10	flagellar assembly protein A
Gth_000673	-2.58	2.52	STAS domain-containing protein
Gth_000764	-3.70	4.07	NAD(P)/FAD-dependent oxidoreductase
Gth_000804	-2.21	2.08	DHA2 family efflux MFS transporter permease subunit
Gth_000832 (*glnA*)	-3.79	3.39	type I glutamate-ammonia ligase
Gth_001034	-3.25	2.38	MerR family transcriptional regulator
Gth_001038	-4.69	4.47	nitrate/nitrite transporter
Gth_001040 (*nirD*)	-5.03	3.73	nitrite reductase small subunit NirD
Gth_001041 (*nirB*)	-5.59	5.53	nitrite reductase large subunit NirB
Gth_001091 (*ureG*)	-4.66	3.90	urease accessory protein UreG
Gth_001093 (*ureE*)	-5.07	5.29	urease accessory protein UreE
Gth_001094 (*ureC*)	-4.87	4.53	urease subunit alpha
Gth_001096 (*ureA*)	-4.91	5.44	urease subunit gamma
Gth_001097 (*urtE*)	-4.69	4.66	urea ABC transporter ATP-binding subunit UrtE
Gth_001098 (*urtD*)	-4.85	5.29	urea ABC transporter ATP-binding protein UrtD
Gth_001099 (*urtC*)	-4.77	5.69	urea ABC transporter permease subunit UrtC
Gth_001100 (*urtB*)	-4.17	4.15	urea ABC transporter permease subunit UrtB
Gth_001101 (*urtA*)	-3.41	2.94	urea ABC transporter substrate-binding protein
Gth_001092	-5.22	5.29	urease accessory protein UreF
Gth_001095	-4.69	4.48	urease subunit beta
Gth_001102	-3.93	3.75	methyl-accepting chemotaxis protein
Gth_001103	-3.13	4.40	GntP family permease
Gth_001135 (*shc*)	2.56	2.32	squalene-hopene cyclase
Gth_001347 (*dinG*)	-2.79	2.73	ATP-dependent DNA helicase DinG
Gth_001350 (*panB*)	-2.87	2.30	3-methyl-2-oxobutanoate hydroxymethyltransferase
Gth_001758	-2.24	2.24	hypothetical protein
Gth_001759	-3.27	3.81	hypothetical protein
Gth_001760	-2.48	2.14	hypothetical protein
Gth_001761	-3.33	2.23	PTS ascorbate transporter subunit IIC
Gth_001768	-3.75	5.73	hypothetical protein
Gth_001771	-3.07	4.91	helix-turn-helix transcriptional regulator
Gth_001941 (*modA*)	-4.85	5.38	molybdate ABC transporter substrate-binding protein
Gth_002015	-2.58	2.19	acetate kinase
Gth_002337 (*fliS*)	-2.36	3.12	flagellar export chaperone FliS
Gth_002338	-2.25	2.10	flagellar hook-associated protein 2
Gth_002356	-2.26	3.48	6-hydroxymethylpterin diphosphokinase MptE-like protein
Gth_002357	-2.25	3.73	UDP-N-acetylglucosamine 4,6-dehydratase family protein
Gth_002358	-2.14	2.18	hypothetical protein
Gth_002359	-3.80	7.00	flagellin
Gth_002369	-2.50	3.82	flagellin

Gth_002370 (*fliS*)	-2.33	2.36	flagellar export chaperone FliS
Gth_002371	-2.34	3.10	EscU/YscU/HrcU family type III secretion system export apparatus switch protein
Gth_002372	-2.54	3.39	hypothetical protein
Gth_002380	-4.72	9.00	SH3 domain-containing protein
Gth_002381	-4.98	10.83	SH3 domain-containing protein
Gth_002382	-3.39	6.12	SpoIID/LytB domain-containing protein
Gth_002444	-5.55	8.38	carbohydrate ABC transporter permease
Gth_002445	-5.05	6.43	carbohydrate ABC transporter permease
Gth_002446	-5.47	7.60	ABC transporter substrate-binding protein
Gth_002679	-2.35	2.15	sucrose-specific PTS transporter subunit IIBC
Gth_003022	-3.41	3.55	NCS2 family permease
Gth_003234	-4.96	3.32	16S ribosomal RNA
Gth_003595	-4.02	4.07	hypothetical protein
Gth_003597	-3.05	3.39	ISLre2 family transposase

*Loci refer to GenIDs in the reference genome available at NCBI under the accession number PRJEB104702

Table S9: Upregulated genes during growth of *G. thermoleovorans* BDO on glucose compared to the wild type on glucose. A color code represents the values of $\log_2$ fold change and $-\log_{10}$(FDR p-value) with stepwise increasing darkness of the color for the following levels: 0-3.99; 4-7.99; 8-11.99; 12 -16

Locus*	$\log_2$foldchange	$-\log_{10}$(FDR p-value)	Function
Gth_000058	3.86	3.17	hypothetical protein
Gth_000066	5.64	3.17	DUF1657 domain-containing protein
Gth_000310 (*spoIIGA*)	2.14	2.42	sigma-E processing peptidase SpoIIGA
Gth_000448 (*dpaA*)	3.89	3.17	dipicolinic acid synthetase subunit A
Gth_000449	4.13	2.93	dipicolinate synthase subunit B
Gth_000964	3.70	2.07	DUF4183 domain-containing protein
Gth_001191	3.72	2.48	TRAP transporter substrate-binding protein
Gth_001192	4.81	4.79	sugar phosphate isomerase/epimerase family protein
Gth_001193	4.54	4.20	thiamine pyrophosphate-binding protein
Gth_001194	4.31	3.25	alcohol dehydrogenase catalytic domain-containing protein
Gth_001195	4.48	3.72	aldehyde dehydrogenase family protein
Gth_001196	4.15	3.05	citryl-CoA lyase
Gth_001246	3.26	2.81	hypothetical protein
Gth_001247	2.72	2.79	hypothetical protein
Gth_001248	3.03	3.17	SIS domain-containing protein
Gth_001249	3.09	3.36	KDGP aldolase
Gth_001250	3.24	3.36	creatininase family protein
Gth_001251	3.09	3.04	phosphotriesterase
Gth_001253	3.42	5.20	PTS ascorbate transporter subunit IIC
Gth_001254	3.82	4.79	PTS sugar transporter subunit IIB
Gth_001255	3.74	4.61	PTS sugar transporter subunit IIA
Gth_001256	3.73	4.79	transcription antiterminator
Gth_001284	2.32	2.96	MaoC/PaaZ C-terminal domain-containing protein
Gth_001409	3.62	3.25	spore coat associated protein CotJA
Gth_001410	3.27	2.02	spore coat protein CotJB
Gth_001411	2.75	2.14	manganese catalase family protein
Gth_001880 (*spoVID*)	2.61	2.60	stage VI sporulation protein D
Gth_001908	3.56	2.25	LuxR C-terminal-related transcriptional regulator
Gth_002260	3.53	2.48	manganese catalase family protein
Gth_002261	3.84	2.56	YuzF family protein
Gth_002466 (*rbsB*)	4.03	2.31	ribose ABC transporter substrate-binding protein RbsB
Gth_002467 (*rbsC*)	4.25	2.96	ribose ABC transporter permease
Gth_002468	4.12	2.66	sugar ABC transporter ATP-binding protein
Gth_002469 (*rbsD*)	3.84	2.25	D-ribose pyranase
Gth_002470 (*rbsK*)	3.60	2.47	ribokinase
Gth_002573 (*spoIID*)	2.75	2.42	sporulation transcriptional regulator SpoIIID
Gth_002679	3.01	2.02	sucrose-specific PTS transporter subunit IIBC
Gth_002960	4.11	2.48	S8 family serine peptidase
Gth_003131	3.05	2.35	xanthine dehydrogenase family protein molybdopterin-binding subunit
Gth_003134	3.03	2.60	hypothetical protein
Gth_003135	2.89	2.02	molybdopterin oxidoreductase family protein
Gth_003136	3.38	2.82	hypothetical protein
Gth_003279	3.61	2.56	CBS domain-containing protein
Gth_003410	4.71	2.51	spore germination protein
Gth_003411	4.30	2.25	spore germination protein GerPB
Gth_003412	2.58	2.02	spore germination protein GerPC
Gth_003414	2.68	2.04	spore germination protein GerPE
Gth_003460	2.99	3.17	Cof-type HAD-IIB family hydrolase
Gth_003463	3.16	2.06	YheC/YheD family protein
Gth_003472	4.56	3.44	small, acid-soluble spore protein, alpha/beta type
Gth_003497	2.94	2.94	SpoVR family protein
Gth_003505	2.87	2.31	hypothetical protein
Gth_003132 (*pucB*)	2.79	2.04	xanthine dehydrogenase accessory protein PucB

*Loci refer to GenIDs in the reference genome available at NCBI under the accession number PRJEB104702

Table S10: Downregulated genes during growth of *G. thermoleovorans* BDO on glucose compared to the wild type on glucose. A color code represents the values of log_2 fold change and $-log_{10}$(FDR p-value) with stepwise increasing darkness of the color for the following levels: 0-|3.99|; |4|-|7.99|; |8|-|11.99|; |12|-|16|

Locus*	Log_2 fold change	$-log_{10}$(FDR p-value)	Function
Gth_000646	-6.50	5.28	ammonium transporter
Gth_000647	-6.62	5.28	DUF294 nucleotidyltransferase-like domain-containing protein
Gth_000648	-6.27	4.79	3'-5' exonuclease
Gth_001038	-4.28	2.60	nitrate/nitrite transporter
Gth_001040 (*nirD*)	-4.66	2.62	nitrite reductase small subunit NirD
Gth_001041 (*nirB*)	-5.57	4.11	nitrite reductase large subunit NirB
Gth_001090	-4.53	3.35	urease accessory protein UreD
Gth_001091 (*ureG*)	-4.60	3.25	urease accessory protein UreG
Gth_001092	-4.80	3.45	urease accessory protein UreF
Gth_001093 (*ureE*)	-4.87	3.91	urease accessory protein UreE
Gth_001094 (*ureC*)	-4.68	3.17	urease subunit alpha
Gth_001095	-4.47	3.34	urease subunit beta
Gth_001096 (*ureA*)	-4.84	3.45	urease subunit gamma
Gth_001097 (*urtE*)	-4.63	3.59	urea ABC transporter ATP-binding subunit UrtE
Gth_001098 (*urtD*)	-4.58	3.68	urea ABC transporter ATP-binding protein UrtD
Gth_001099 (*urtC*)	-4.55	3.44	urea ABC transporter permease subunit UrtC
Gth_001100 (*urtB*)	-4.36	3.78	urea ABC transporter permease subunit UrtB
Gth_001101 (*urtA*)	-3.87	3.34	urea ABC transporter substrate-binding protein
Gth_001102	-3.61	2.33	methyl-accepting chemotaxis protein
Gth_001314	-5.80	2.20	16S ribosomal RNA
Gth_001941 (*modA*)	-3.68	2.11	molybdate ABC transporter substrate-binding protein

*Loci refer to GenIDs in the reference genome available at NCBI under the accession number PRJEB104702

Table S11: Mutations identified by WGS of the strain *G. thermoleovorans* AA after ALE on 45 mM BDO

Affected locus*	Putative function	Mutation (position in genome)**	Putative effect
IGR of Gth_000113-Gth_000114	C39 family peptidase-asparagine synthase	100834_SNV_C_A	mutation in predicted promoter
Gth_000361	IS701 family transposase	346223_SNV_C_T	silent mutation A379A
IGR of Gth_000433-Gth_000434	PolC-type DNA polymerase III-ribosome maturation factor RimP	415874_SNV_C_A	alteration of a regulatory region
IGR of Gth_000711-Gth_000712	YitT family protein	713116_SNV_C_T	silent mutation L36L
IGR of Gth_000873-Gth_000874	glucose 1-dehydrogenase-glucose 1-dehydrogenase	858331_SNV_G_A	alteration of a regulatory region
Gth_001074	IS481 family transposase	1060061_SNV_G_T	silent mutation S185S
Gth_01074	IS481 family transposase	1060719_SNV_G_A	A405T
Gth_001075	AAA family ATPase	1060858_SNV_G_A	V38M
Gth_001075	AAA family ATPase	1060890_SNV_C_T	silent mutation I48I
Gth_001075	AAA family ATPase	1060989_SNV_A_G	silent mutation G81G
IGR of Gth_001082-Gth_001083	aldehyde dehydrogenase family protein-cyclase family protein	1069449_SNV_G_A	alteration of a regulatory region
Gth_001270	hypothetical protein	1254061_SNV_G_T	L732F
IGR of Gth_001402 (*sleB*)-Gth_001403 (*prsW*)	spore cortex-lytic enzyme-glutamic-type intramembrane protease PrsW	1377817_SNV_G_A	alteration of a regulatory region
Gth_003068	AAA family ATPase	2967653_SNV_C_G	silent mutation R173R
Gth_003297	AAA family ATPase	3183207_SNV_G_A	R217A
Gth_003304	hypothetical protein	3190655_SNV_G_A	L218F
Gth_003335	hypothetical protein	3222587_SNV_G_A	silent mutation E90E
Gth_003335	hypothetical protein	insertion 79 bp (3222710)	frameshift, loss of function
Gth_003371	alcohol dehydrogenase AdhP	3254310_SNV_G_A	silent mutation E69E

IGR of Gth_003498-Gth_003499	hypothetical protein-response regulator	3386760_SNV_C_T	alteration of a regulatory region
Gth_003607	hypothetical protein	3485513_SNV_G_A	D183N
IGR of Gth_003645-Gth_003646	hypothetical protein-DUF6431 domain-containing protein	3523406_SNV_C_T	alteration of a regulatory region
Gth_003648	IS701 family transposase	3524776_SNV_A_C	silent mutation V276V
Gth_003648	IS701 family transposase	3524788_SNV_G_C	silent mutation L272L
Gth_003648	IS701 family transposase	3524823_SNV_C_T	V263I
Gth_003648	IS701 family transposase	3524833_SNV_C_G	silent mutation R259R
Gth_003648	IS701 family transposase	3524857_SNV_G_A	silent mutation D249D
Gth_003648	IS701 family transposase	3524881_SNV_T_C	silent mutation E241E
Gth_003648	IS701 family transposase	3524893_SNV_A_G	silent mutation R237R
Gth_003648	IS701 family transposase	3524899_SNV_A_G	silent mutation A235A
Gth_003648	IS701 family transposase	3524902_SNV_A_G	silent mutation Y234Y
Gth_003648	IS701 family transposase	3524932_SNV_C_T	silent mutation E224E

*Affected loci and **positions refer to gene-IDs in the reference genome available at ENA under the accession number PRJEB104702

Table S12: Mutations identified by WGS after ALE of *G. thermoleovorans* AA on 15 mM AA

Affected locus*	Putative function	Mutation (position in genome)**	Putative effect
IGR of Gth_000225 - Gth_000226	glycosyltransferase; YkyB family protein	218642_SNV_C_T	alteration of regulatory region
IGR of Gth_000271 - Gth_000272	DUF420 domain-containing protein; Asp23/Gls24 family envelope stress response protein	insertion at 260691	alteration of regulatory region
Gth_000361	IS701 family transposase	346223_SNV_C_T	silent mutation A379A
IGR of Gth_000849 - Gth_000850	NAD-dependent protein deacylase; acyl-CoA dehydrogenase family protein	837815_SNV_C_T	alteration of regulatory region
IGR of Gth_001774 - Gth_001776	ImmA/IrrE family metallo-endopeptidase; YrzI family small protein	1689977_SNV_T_C	alteration of regulatory region
Gth_003635	MvaI/BcnI family restriction endonuclease	deletion 3514644-3514665	frameshift, loss of function

*Affected loci and **positions refer to gene-IDs in the reference genome available at ENA under the accession number PRJEB104702
